# In vivo evaluation of the anti-inflammatory efficacy of liposomes loaded with *Echinacea purpurea* roots extracts

**DOI:** 10.1007/s13346-026-02109-6

**Published:** 2026-04-09

**Authors:** Sara F. Vieira, Diana Fonseca-Rodrigues, Daniel Mendanha, Vânia I. B. Castro, Ricardo A. Pires, Rui L. Reis, Filipa Pinto-Ribeiro, Helena Ferreira, Nuno M. Neves

**Affiliations:** 1 3B’s Research Group, I3BS – Research Institute on Biomaterials, Biodegradables and Biomimetics, University of Minho, Headquarters of the European Institute of Excellence On Tissue Engineering and Regenerative Medicine, AvePark, Parque de Ciência E Tecnologia, Rua Ave 1, Edifício 1 (Sede), 4805-694 Barco Guimarães, Portugal; 2 ICVS/3B’s–PT Government Associate Laboratory, Braga/Guimarães, Portugal; 3 Life and Health Sciences Research Institute (ICVS), School of Health Sciences, University of Minho, Campus de Gualtar, 4710-057 Braga, Portugal

**Keywords:** *Echinacea purpurea* roots extracts, Alkylamides, Liposomes, Synovial inflammation, Animal model

## Abstract

WHO ranked chronic inflammatory diseases as the most significant cause of death. The current therapeutic approach is the constant administration of clinically available anti-inflammatory drugs; however, this strategy presents severe side effects. New entities derived from plants have shown their potential for developing safe and effective therapies. Particularly, *Echinacea purpurea* extracts have demonstrated strong anti-inflammatory properties. Therefore, dichloromethanolic extracts of *E. purpurea* roots (DE-R), rich in alkylamides, were used to produce an anti-inflammatory formulation. To increase therapeutic efficacy, DE-R were loaded into liposomes (LPs), which were also engineered to actively target M1 macrophages via surface functionalization with folic acid (FLPs). The developed formulations were homogeneous, with a mean particle size of ≈114 nm. The liposomes were cytocompatible at all tested concentrations and strongly reduced IL-6 production in LPS-stimulated macrophages. As expected, FLPs + DE-R were 6.3 times better than LPs + DE-R, and 9.7 times greater than free DE-R. The anti-inflammatory activity of free DE-R and DE-R-loaded FLPs was assessed in an experimental rat model of inflammation. A single intra-articular injection of the FLPs + DE-R promoted a significant reduction of edema, inflammatory pain, and immune cell infiltration, particularly of CD68^+^ macrophages, as well as IL-6 expression. This formulation reduced synovial inflammation more efficiently than free DE-R. We also demonstrated that the developed formulation was safe, as no welfare changes or harmful toxicity to major organs were observed. Therefore, *E. purpurea* roots extracts loaded in liposomes can be used as a natural, innovative, and powerful new formulation to treat chronic inflammatory diseases.

**Graphical Abstract:**

**Figure:**
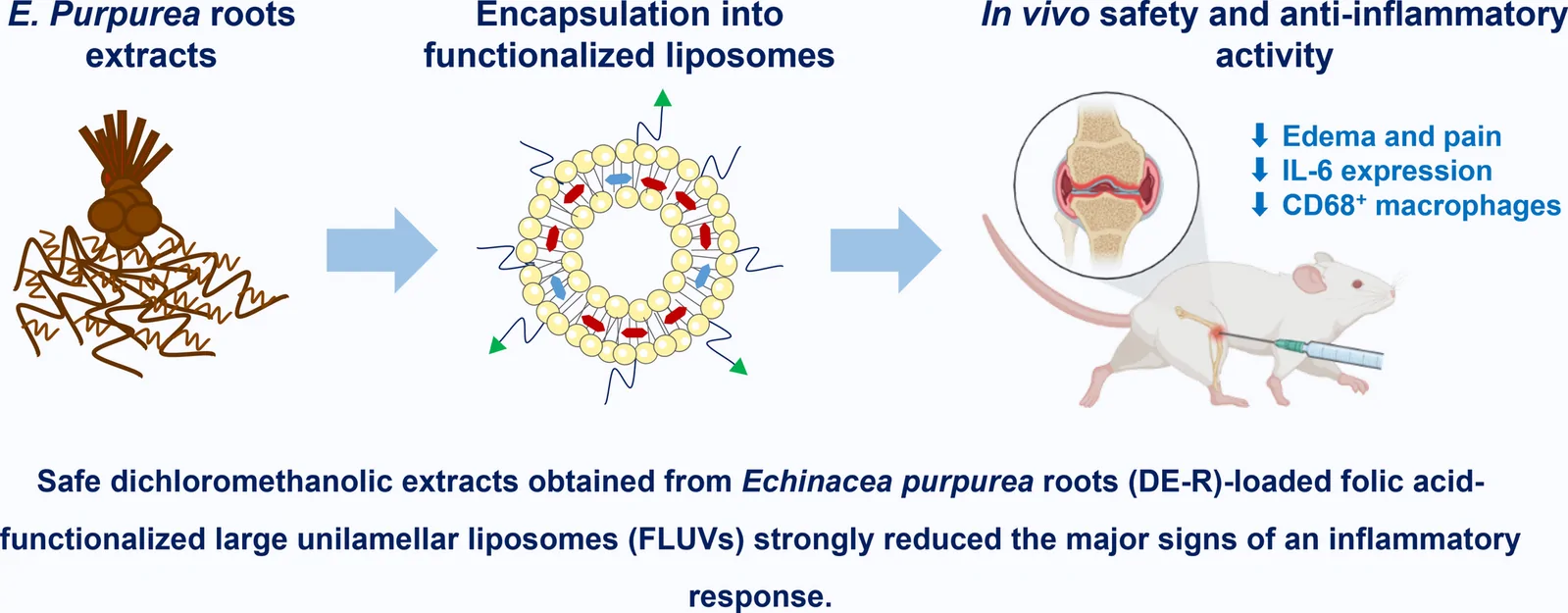

**Supplementary Information:**

The online version contains supplementary material available at https://doi.org/10.1007/s13346-026-02109-6.

## Introduction

Chronic inflammation is a long-term process that may last weeks, months, or even a lifetime [1], and whose prevalence is estimated to increase over the next few decades [2]. More than half of the global deaths are correlated with chronic inflammatory diseases (e.g., arthritic diseases, stroke, chronic respiratory diseases, heart disorders, and type 1 and 2 diabetes) [3] and, consequently, WHO ranked chronic inflammatory diseases as the most significant cause of death in the world nowadays [4, 5].

One of the chronic inflammation-mediated diseases with a higher incidence is arthritis [6, 7]. Arthritic diseases affect more than 350 million people in the world and are often associated with extremely high societal and economic burdens, being a leading cause of work disability [8]. They comprise more than 150 different joint disorders, with the most common being osteoarthritis and rheumatoid arthritis. These inflammatory diseases usually affect the hands, hips, spine, and/or knees, with the main symptoms being pain, warmth, swelling, stiffness, and decreased range of flexibility in or around the joints [9]. They are complex diseases characterized by pathological changes in the joint tissues, including cartilage, subchondral bone, ligaments, meniscus, and the synovial membrane [10], with a typical hallmark being the development of low-grade synovial inflammation (synovitis) [11].

Synovitis is characterized by the continuous recruitment of CD68^+^ macrophages and the proliferation of fibroblast-like synoviocytes, resulting in hyperplasia of the synovial lining [12, 13]. Additionally, the underlying tissue (subintima) is heavily infiltrated with T and B lymphocytes, plasma cells, and macrophages, being associated with stromal edema and proliferation of blood vessels [13]. Activated macrophages, strongly expressing the folate receptor on their surface, are the most abundant immune cells in the synovial membrane and have been correlated with arthritis severity and symptoms [14]. Indeed, activated macrophages in the synovium contribute to articular degeneration due to the higher production of pro-inflammatory cytokines (e.g., interleukin – IL – 6, IL-1β, and tumor necrosis factor – TNF – α) and reactive oxygen and nitrogen species (ROS/RNS) [15, 16]. Consequently, the disruption of macrophage infiltration, as well as the amelioration of synovial inflammatory mediator production have been proposed as a promising therapeutic approach.

Currently, there is no cure for arthritic diseases, and the main goals of current treatments are to reduce pain and improve joint function. Symptoms are usually treated with a combination of therapies. For instance, physical therapy with co-administration of painkillers (e.g., acetaminophen) and/or nonsteroidal anti-inflammatory drugs (NSAIDs, e.g., diclofenac and celecoxib) [17]. More potent drugs, such as corticosteroids (e.g., dexamethasone), conventional disease-modifying antirheumatic drugs (cDMARDs; e.g., methotrexate), and biologic disease-modifying antirheumatic drugs (bDMARDs, e.g., TNF or IL-6 inhibitors) can also be prescribed [18, 19]. Nonetheless, anti-inflammatory drugs are associated with severe side effects, mainly if administered for long periods (e.g., gastrointestinal, cardiovascular, blood, renal, and adrenal injuries), and their use is limited [20, 21]. In addition, immunosuppressant agents increase the risk of infection and/or cancer [22]. Therefore, newer, safer drugs are urgently needed to effectively treat the inflammatory process.

Plant-derived drugs are potential alternatives for the safe and effective management of inflammation [23], as natural products account for about one-quarter of all new drugs approved for clinical use, of which almost half are derived from plants [24]. Recently, *Echinacea purpurea* extracts demonstrated strong anti-inflammatory properties [25–28]. Particularly, dichloromethanolic extracts obtained from *E. purpurea* roots (DE-R) demonstrated the strongest anti-inflammatory activity in the presence of lipopolysaccharides (LPS)-stimulated macrophages. Indeed, these extracts significantly reduced the production of pro-inflammatory mediators (IL-6, IL-1β, TNF-α, ROS/RNS) [25].

The main active principles in DE-R are alkylamides [25], however, due to their low aqueous solubility, a suitable drug delivery approach should be used to improve their biodistribution. The encapsulation of drugs in a colloidal delivery system could be an efficient strategy to improve their pharmacokinetics [29]. Indeed, in addition to protecting them from degradation, these systems increase the therapeutic index of these drugs. To improve the blood circulation time, the surface of nanocarriers can be coated with hydrophilic polymers, such as polyethylene glycol (PEG), to provide a protective layer that avoids aggregation, opsonization, and phagocytosis [30]. Moreover, nanocarriers exploit the enhanced permeability and retention (EPR) effect at sites of inflammation [31]. In addition to passive targeting, nanocarriers can be functionalized with a specific moiety to achieve active targeting. Considering the specific targets present at the pathological site, it is possible to modify the nanocarrier surface with a specific ligand (e.g., antibody, protein, peptide, or carbohydrate) to enable their accumulation in the diseased tissue. For example, folic acid is used as a targeting moiety for the folate receptors on activated macrophages at the inflammatory sites [32]. Consequently, passive and active targeting, which promote nanocarrier accumulation at the desired site, will reduce the amount of drug required to achieve a therapeutic effect and reduce its side effects.

Among nanocarriers, liposomes are the safest, most effective, and versatile drug delivery systems [33]. Indeed, due to their advantages, liposomes have been successfully translated into the clinic for the treatment of different diseases [34]. Liposomes are composed of phospholipids, the main components of the cell membrane, and, consequently, display excellent biocompatibility [35]. Liposomes can encapsulate lipophilic, amphipathic, and/or hydrophilic drugs. A wide variety of phospholipids is available for liposome production, ranging from natural to synthetic ones [35]. Among them, phosphatidylcholine (PC), derived from egg yolk, was widely used and shows anti-inflammatory and antioxidant activities [36]. Additionally, PC is the major membrane phospholipid in eukaryotic cells [37]. Liposomes can also be used for intra-articular (IA) administration, the most effective route for treating joint diseases [38], as they allow for the delivery of high drug concentrations directly into the joint cavity.

This work describes the development of folic acid-functionalized liposomes loaded with DE-R, further used as an IA drug delivery system for the treatment of arthritic diseases. After its production and characterization, cytocompatibility and anti-inflammatory activity of the liposomes were investigated using LPS-stimulated macrophages. The ability of pro-inflammatory macrophages to internalize LPs and FLPs was also assessed. Then, the in vivo safety and efficacy of the developed formulation were evaluated in a carrageenan-induced early inflammation rat model.

## Materials and methods

### Materials

*Echinacea purpurea* L. (purple coneflower) was purchased from Cantinho das Aromáticas (Vila Nova de Gaia, Portugal) in May 2017. The plants were immediately transferred to the soil and grown according to sustainable agriculture practices (41°37′04.5″ N, 7°16′14.4″ W). After three years of cultivation, the roots, including rhizomes, were harvested in the autumn (November 2021). The plants were subsequently dried in the dark and stored at room temperature (RT) protected from the light.

Ethanol (EtOH) and dichloromethane (DCM) were obtained from Fisher Scientific, Portugal. Ultra-pure water was obtained from a Milli-Q^®^ Direct Water Purification System (Milli-Q Direct 16, Millipore). Acetonitrile (ACN, HPLC grade), methanol (HPLC grade), formic acid (99%, analytical grade), phosphate-buffered saline (PBS), phorbol 12-myristate 13-acetate (PMA), LPS (*Escherichia coli*, O26:B6), egg yolk L-α-phosphatidylcholine (EPC), disposable PD 10 desalting columns (Sephadex G-25 M column), Roswell Park Memorial Institute (RPMI)−1640 medium, dexamethasone, Triton X-100, λ-carrageenan, sodium citrate tribasic dihydrate, Tween 20, bovine serum albumin (BSA), and high-purity standards of echinacoside, chicoric acid, caftaric acid, caffeic acid, chlorogenic acid, and cynarin were obtained from Sigma-Aldrich, Portugal. Echinacea isobutylamide standards kit, composed of undeca-2E/Z-ene-8,10-diynoic acid isobutylamide, dodeca-2E-ene-8,10-diynoic acid isobutylamide, and dodeca-2E,4E-dienoic acid isobutylamide, was acquired from ChromaDex, Los Angeles, CA, USA. High-purity standard dodeca-2E,4E,8Z,10E/Z-tetraenoic acid isobutylamide was obtained from Biosynth Carbosynth, Spain. Fetal bovine serum (FBS), antibiotic/antimycotic solution, Dulbecco’s phosphate-buffered saline (DPBS), formalin 10% (*v/v*), GlutaMAX™-I CTS™, HEPES (1 M), Richard-Allan decalcifying solution, paraffin, hematoxylin, mouse monoclonal CD68 Monoclonal Antibody (MA5-13324), and Alexa Fluor 594 donkey anti-mouse IgG were purchased from Thermo Fisher Scientific, Portugal. Dimethyl sulfoxide (DMSO) was obtained from VWR, Portugal. Human IL-6 DuoSet Enzyme-linked immunosorbent assay (ELISA) kits and DuoSet ELISA Ancillary Reagent Kit 2 were purchased from R&D Systems, Minneapolis, USA. AccuClear^®^ Ultra High Sensitivity dsDNA Quantitation was purchased from Biotium, Fremont, CA, USA. Deep Blue Cell Viability™ Kit was obtained from BioLegend, San Diego, CA, USA. LabAssay Phospholipid was acquired from FUJIFILM Wako Shibayagi Corporation, Japan. Mouse monoclonal anti-IL6 antibody (ab9324) was purchased from abcam, Cambridge, UK. DAPI (4′,6-diamidino-2-phenylindole) was purchased from Biotium, Fremont, CA, USA. Rhodamine phalloidin was acquired from cytoskeleton, Denver, CO. Celecoxib was obtained from abcr GmbH, Karlsruhe, Germany. 1,2-distearoyl-sn-glycero-3-phosphoethanolamine-N-[amino(polyethylene glycol)−2000] ammonium salt (DSPE-PEG), 1,2-distearoyl-sn-glycero-3-phosphoethanolamine-N-[folate(polyethylene glycol)−2000] ammonium salt (DSPE-PEG-Folate), polycarbonate membranes (1.0, 0.4, 0.2, and 0.1 μm), and filter support were obtained from Avanti Polar Lipids, Inc, Alabama, USA. 22-(N-(7-nitrobenz-2-oxa-1,3-diazol-4-yl)amino)−23,24-bisnor-5-cholen-3βol (NBD cholesterol) was obtained from Molecular Probes, Eugene, OR, USA. Trypsin/EDTA was purchased from Corning, Arizona, USA. Wistar rats were purchased from Charles Rivers, Saint Germain Nuelles, France. Coffee filter paper N4 was acquired in a local supermarket. VECTASHIELD^®^ Antifade Mounting Medium was obtained from Vector Laboratories, Burlingame, USA. Eosin G or Y 0.5% alcoholic was acquired from Diapath, Martinengo, Italy. Xylene was purchased from CARLO ERBA Reagents, Chaussée du Vexin, France.

### Bioactive compounds extraction

Dried *E. purpurea* roots and rhizomes were ground in an ultra-centrifugal mill (Retsch GmbH, Haan, Germany) at 16,000 rpm just before extraction. DE-R were prepared by stirring 120 g of the sample in 1200 mL of DCM at RT for 24 h in the dark. The DCM was changed after 12 h of the extraction process. The combined solutions were filtered through a N4 coffee filter paper. Then, samples were concentrated by DCM evaporation at RT under reduced pressure using a rotary evaporator (IKA VACSTAR D S099, IKA^®^-Werke, Germany). After collecting the concentrated DE-R solutions into an amber vial, the remaining organic solvent was removed under nitrogen gas. The dried extract (extraction yield of 2.02%, *w/w*) was stored at − 80 °C until further assays.

### Chemical characterization of DE-R

The chemical characterization of DE-R was performed using a liquid chromatography-high-resolution mass spectrometry (LC-HRMS) method previously described by our research group [25]. Briefly, DE-R was dissolved in methanol at 2.0 mg/mL. Then, the DE-R solution was centrifuged, filtered through a 0.2 μm filter, and injected into the UltiMate 3000 Dionex ultra-high-performance liquid chromatography (UHPLC, Thermo Scientific, Lisbon, Portugal), coupled to an ultrahigh-resolution quadrupole–quadrupole time-of-flight (UHR–QqTOF) mass spectrometer (Impact II, Bruker). Bruker Compass DataAnalysis 5.1 software (Bruker) was used to analyze LC-HRMS-acquired data. The identification of the compounds present in DE-R was confirmed by the matching of the retention time (t_R_, min), the mass-to-charge ratio (*m/z*) of the molecular ion, and MS/MS fragmentation patterns with the standards (Supplementary Table S1). If the t_R_ and MS data of a compound did not match with those of available standards, the potential identity of it was assigned by comparing the MS/MS spectrum with the theoretical data of MS/MS fragments and information obtained from the literature [39–45].

### Liposomes preparation

The liposomes were prepared using the thin-film hydration method followed by extrusion [46, 47]. Liposomes (33 mM) were prepared by mixing EPC (15 mg/mL) and DSPE-PEG (18 mg/mL), both dissolved in ethanol, at a molar ratio of 0.85:0.15. The organic solvent was then removed using a rotavapor (IKA VACSTAR D S099, IKA^®^-Werke, Germany) to form a lipid film. To eliminate the organic solvent residues, the lipid film was put under a stream of nitrogen gas for more than 3 h. After adding PBS to hydrate the lipid film, the mixture with glass beads was vortexed (Vortex 3, IKA^®^-Werke, Germany) for 15 min to produce multilamellar liposomes (MLVs). The MLVs suspension rested for more than 15 min. Then, it was sequentially extruded (Mini Extruder, Avanti Polar Lipids, Inc.) through polycarbonate membranes with pore sizes of 1000 nm (10 times), 400 nm (10 times), 200 nm (32 times), and 100 nm (1 time) to produce LPs.

To obtain LPs functionalized with folic acid (FLPs), 1.5% (*n/n*) DSPE-PEG-Folate (20 mg/mL) [46] was added to the previously referred lipidic solution. For the internalization studies, 1% (*n/n*) NBD-cholesterol in ethanol (10 mg/mL) was also mixed with these lipid solutions [46]. DE-R was encapsulated in both LPs and FLPs by adding the extract, dissolved in DCM (750 mg/mL), to the organic solution prior to lipid film formation, resulting in LPs + DE-R and FLPs + DE-R, respectively. To remove the non-entrapped DE-R, LPs + DE-R, and FLPs + DE-R suspensions were subjected to column chromatography, using a Sephadex G-25 M column. The DE-R-loaded liposome suspensions were collected into a sterile vial protected from the light. Finally, all the formulations were sterilized with a 0.2 μm filter and stored at 4 °C until further analysis. In each step of liposome preparation, a volume (50 μL) was collected to quantify the amount of alkylamides 8/9 by high-performance liquid chromatography (HPLC, see Section Entrapment efficiency). The amount of DE-R used in the formulations was selected based on preliminary optimization studies, taking into account its maximum solubility in DCM and its impact on encapsulation efficiency. Based on these calculations, the theoretical concentration of DE-R in the lipid film was 8125 µg/mL. This value was then used to calculate the percentage of alkylamides 8/9 entrapped in the liposomes by interpolation from HPLC measurements.

### PC quantification

The PC concentration of LPs, FLPs, LPs + DE-R, and FLPs + DE-R was determined using the LabAssay Phospholipid kit, according to the manufacturer’s instructions. Shortly thereafter, choline chloride standards ranging from 0 to 7.76 × 10^–3^ mol/L were prepared in distilled water. Then, the blank (PBS), liposome samples or standards (2 μL) were mixed with the chromogen reagent (300 μL) in a 96-well plate and incubated at 37 °C. After 5 min, a microplate reader (SYNERGY HT, Bio-Tek, Vermont, USA) was used to read the absorbance of the blue pigment formed at 600 nm. The PC concentration (mol/L) of each formulation was calculated from the standard curve relating choline chloride concentration to absorbance.

### Liposomes characterization

#### Size distribution and zeta potential measurement

The LPs, FLPs, LPs + DE-R, and FLPs + DE-R were analyzed by dynamic light scattering (DLS) to evaluate their size and polydispersity index (PDI), using disposable polystyrene cuvettes. The surface electric charge was evaluated using a dip cell and laser Doppler micro-electrophoresis. The measurements were performed at 37 °C in a Zetasizer Nano ZS instrument (Malvern Instruments, Worcestershire, UK). The sterilized samples were diluted in PBS (1:66, *v/v*).

#### Liposomes stability

The stability of LPs, FLPs, LPs + DE-R, and FLPs + DE-R stored in the dark at 4 °C under static and sterilized conditions was evaluated for 90 days through a regular determination of their size, PDI, and zeta potential, as previously described (Section Size distribution and zeta potential measurement).

#### Entrapment efficiency

The isomeric pair dodeca-2E,4E,8Z,10E/Z-tetraenoic acid isobutylamide (alkylamides 8/9 according to the Bauer system [45]) is the most abundant alkylamide in *E. purpurea* [48]. Then, considering the concentration of alkylamides 8/9 in liposomes, the entrapment efficiency (EE) of DE-R was determined according to Eq. (1):

1 $$EE (\%)= \frac{{\left[Alkylamide 8/9 \right]}_{LPs}}{{\left[Alkylamide 8/9\right]}_{MLVs}}\times 100$$

HPLC was used to quantify alkylamides 8/9 in DE-R and liposome formulations. Alkylamides 8/9 standard was prepared in methanol at concentrations of 0, 2.5, 5, 10, 20, 50, and 100 μg/mL. Before chromatographic analysis, DCM was evaporated, and the resulting DE-R residue was dissolved in methanol. MLVs, FMLVs, MLVs + DE-R, FMLVs + DE-R, LPs, FLPs, LPs + DE-R, and FLPs + DE-R were diluted in methanol.

The chromatographic separation of the alkylamides 8/9 standard (100 μg/mL) was first optimized by analytical HPLC, following a previously reported method [49]. An analytical HPLC system (Alliance e2695, Waters, Milford, USA) equipped with a quaternary pump, a variable volume injection loop, a temperature-controlled autosampler, a column oven, and a dual channel wavelength detector (Waters 2489, 190–700 nm) was used. The chromatographic separation was performed on a Zorbax SB-C18 stable bond analytical column (4.6 × 250 mm, 5 μm, Agilent, USA). HPLC control and data management were performed USING Empower 3.0. DE-R, MLVs, FMLVs, MLVs + DE-R, FMLVs + DE-R, LPs, FLPs, LPs + DE-R, FLPs + DE-R, and standards were analyzed using isocratic elution (70% of ACN and 30% water containing 0.1% formic acid) for 15 min, at a flow rate of 1 mL/min. The column was set at RT. The injection volume was 20 μL. The UV spectra were acquired at 254 nm. Alkylamides 8/9 were identified by comparison of its t_R_ (6.2 min) with that of the standard. Alkylamides concentration (μg/mL) in each sample was calculated using the standard curve relating to the alkylamide standard concentration and the peak absorbance. Representative chromatograms of the tested samples for alkylamides 8/9 quantification (Figure S1) show that liposomes did not interfere with alkylamides quantification.

### In vitro studies

#### Cell seeding

The anti-inflammatory activity of DE-R, LPs, FLPs, LPs + DE-R, and FLPs + DE-R was evaluated using a human peripheral blood monocyte cell line (THP-1) obtained from the American Type Culture Collection (ATCC^®^ TIB-202™), as previously described [50]. THP-1 cell line, at passages 13–15, was cultured in complete RPMI (cRPMI, RPMI 1640 medium supplemented with 10% FBS, 1% antibiotic/antimycotic solution, 1% HEPES, 1% glutamax), at 37 °C in a humidified atmosphere of 5% CO_2_. Briefly, 5 × 10^5^ cells were seeded in adherent 24-well culture plates and treated with PMA (100 nM) for 24 h. Then, the non-attached cells were removed by pipette aspiration, and the adherent cells were washed twice with warm cRPMI medium. After 48 h, the cells were stimulated with LPS (100 ng/mL) in fresh cRPMI. After 2 h, without removing LPS, the DE-R (dissolved in DMSO) and liposomal formulations containing the extract were added at different concentrations (0, 0.5, 1.5, 2.5, 3.5, 5, and 10 μg/mL of alkylamides 8/9) by their dilution in cRPMI. LPs and FLPs at final concentrations of 0.125, 0.25, 0.50, 1.0, 1.5, and 2.0 mM of PC in the well were also evaluated. After 22 h of culture, the cells’ metabolic activity (see Section Metabolic activity), and DNA content (see Section DNA quantification) were determined. Immediately before, the medium was harvested (triplicates were mixed and homogenized) and stored in aliquots at − 80 °C until cytokine quantification (see Section IL-6 quantification). LPS-stimulated macrophages cultured without treatment were used as a positive control of cytokine production. Dexamethasone and celecoxib, both at 10 µM [51, 52], were used as positive controls of compounds having anti-inflammatory activity. Negative controls (cells without LPS; no stimulation) were also evaluated. Controls containing the same percentage of DMSO in the maximal concentration of extracts were also tested and did not affect the cell viability.

#### Metabolic activity

The metabolic activity of LPS-stimulated macrophages incubated with DE-R and liposomal formulations was determined using the Deep Blue Cell Viability™ Kit, according to the manufacturer’s recommendations. Briefly, 400 µL of the working solution (2 mL of Deep Blue solution mixed with 20 mL of cRPMI) was added to the cells, which were incubated at 37 °C in a humidified atmosphere containing 5% CO_2_ for 3 h. The working solution was also added to an empty well as a blank. During this time, live cells convert the blue resazurin to the pink resorufin viametabolic enzymes. The fluorescence (Exc/Em: 530/590 nm) was recorded in triplicate on a microplate reader (SYNERGY HT, Bio-Tek, Vermont, USA). The results of metabolic activity are expressed as a percentage related to the control (non-treated LPS-stimulated macrophages).

#### DNA quantification

The DNA concentration of LPS-stimulated macrophages incubated with or without DE-R incorporated into liposomal formulations was quantified using the AccuClear^®^ Ultra High Sensitivity dsDNA Quantitation kit, performed according to the manufacturer’s recommendations. Briefly, after measuring metabolic activity, the cells were gently washed twice with sterile PBS. Then, 1 mL of ultra-pure water was added to each well, and the plate was frozen at −80 °C until further analysis. After thawing, the samples were transferred to microtubes and sonicated for 15 min. The standard curve was prepared over the range of 0 to 5 µg/mL. The sample or standard (10 µL) was added in triplicate to a black 96-well plate, followed by the addition of the AccuClear^®^ dye working solution (200 µL). The plate was incubated for 5 min in the dark, and the fluorescence (Exc/Em: 485/528 nm) was measured in a microplate reader (SYNERGY HT, Bio-Tek, Vermont, USA). The DNA concentration (µg/mL) of each sample was extrapolated from the standard curve relating DNA concentration to fluorescence intensity. The DNA results are expressed as the relative concentration of the samples divided by the DNA content of the controls (non-treated LPS-stimulated macrophages).

#### IL-6 quantification

The anti-inflammatory effects of DE-R and liposomes on IL-6 production by LPS-stimulated macrophages were evaluated using a commercially available ELISA kit according to the manufacturer’s instructions. The values obtained were normalized to their respective DNA concentration. The results obtained for the determination of the anti-inflammatory activity are expressed in percentages related to the control (non-treated LPS-stimulated macrophages) [53].

#### Liposomes internalization

The uptake of LPs + DE-R and FLPs + DE-R by LPS-stimulated macrophages was evaluated by flow cytometry and confocal analysis. THP-1 seeding and culture were performed as previously described (Section Cell seeding). However, in these assays, THP-1 was seeded at a density of 1.5 × 10^4^ cells/well in 48-well plates or 2.5 × 10^5^ cells/well in 12-well chamber μ-slides (Ibidi, Gräfelfing, Germany) for flow cytometry and confocal analysis, respectively.

To evaluate cellular uptake by flow cytometry, fluorescent-labeled LPs + DE-R and FLPs + DE-R were incubated with LPS-stimulated macrophages for 2, 4, and 22 h, at 37 °C in a humidified 5% CO_2_ atmosphere. At each time point, the medium was removed, and the cells were washed twice with DPBS to remove non-internalized liposomes. Then, 300 μL of trypsin/EDTA was added and incubated for 5 min, at 37 °C in a humidified 5% CO_2_ atmosphere to detach the cells. The bottom was gently scraped, and the cell suspension was collected and transferred to a flow cytometry tube containing 600 μL of cRPMI. After centrifugation (300 g, 5 min), the supernatant was discarded, and the pellet was resuspended and fixed with 250 μL of 4% formalin in DPBS. The samples were stored at 4 °C until analysis. Data were acquired using a flow cytometer (BD FACSCalibur, BD Biosciences, USA) and analyzed with FlowJo Engine V4 software (BD Biosciences, USA). Results are expressed as the mean percentage of cellular uptake from 10,000, and normalized to cells incubated without liposomes to avoid interference from liposome fluorescence.

For confocal analysis, LPS-stimulated macrophages treated with fluorescent-labeled LPs + DE-R and FLPs + DE-R for 4 h were fixed with 150 μL of 10% formalin and stored at 4 °C for further staining and confocal analyses. The fixed cells were permeated with 0.2% (*v/v*) Triton X-100 diluted in PBS for 5 min. Afterward, phalloidin (1:250 in DPBS) was added for 40 min. followed by DAPI (1:1000 in DPBS) for 30 min to stain the cytoskeleton and cell nuclei, respectively. Between all steps, the cells were washed twice for 5 min with DPBS. Images were acquired using excitation wavelengths of 405 nm (DAPI), 488 nm (NBD-cholesterol), and 561 nm (phalloidin) in an Inverted Confocal Microscope with Incubation (TCS SP8, Leica, Mannheim, Germany).

### In vivo studies

#### Animal care and ethical issues

In vivo experiments were performed in 5-week-old male Wistar rats (*n* = 48). Animals were housed two per cage in a limited-access rodent facility, in a climate-controlled room (temperature: 20–24 °C; relative humidity: 55 ± 10%) and a 12/12 h light/dark cycle (starting at 8:00 am). Food and water were provided ad libitum. General health parameters were evaluated twice per week, and body weight was measured weekly. The experimental protocol was approved by the Institutional Ethical Commission and Direção Geral de Alimentação e Veterinária (DGAV; ORBEA EM/ICVS-I3Bs_011/2018). The European Community Council Directives 86/609/EEC and 2010/63/EU were followed concerning the use of animals for scientific purposes. All efforts were made to reduce animal suffering and minimize the number of animals required to produce reliable scientific data.

#### Induction of inflammation

A carrageenan-induced knee inflammation animal model was used to evaluate the therapeutic efficacy of FLPs + DE-R, induced as previously described [54]. The animals were anesthetized by an intraperitoneal injection of a mixture of ketamine (0.75 mg/kg) and medetomidine (0.50 mg/kg). Then, 100 μL of a solution of 3% carrageenan dissolved in sterile saline was injected into the synovial cavity of the right knee joint of each animal. Healthy control animals (Sham) received a 100 μL PBS injection. The left knee joint was used as a control. After the procedure, the anesthesia was reversed with a subcutaneous injection of atipamezole hydrochloride (1 mg/kg), and the animals were monitored until fully awake (eating and grooming).

#### Treatment

After 3 days of the induction of inflammation in male Wistar rats (see Section Induction of inflammation), the animals were treated with an IA injection of the different formulations (50 μL), resulting in six different groups (8 rats/group): *(i)* healthy (Sham), *(ii)* inflammation control, *(iii)* inflammation + FLPs (0.4 μM PC), *(iv)* inflammation + DE-R (3.5 μg/mL alkylamides 8/9), *(v)* inflammation + FLPs + DE-R (0.4 μM PC and 3.5 μg/mL alkylamides 8/9), and *(vi)* inflammation + celecoxib (10 μM). Sham and control animals were injected with a vehicle (PBS). The DE-R, FLPs, FLPs + DE-R, and celecoxib were diluted in the vehicle to achieve their final concentrations.

Disease progression was assessed at days 0, 4, and 10 by measuring knee edema (see Section Evaluation of the knee edema), mechanical allodynia (see Section Mechanical allodynia), and hyperalgesia (see Section Mechanical hyperalgesia). On the 10^th^ day, the animals were euthanized and transcardially perfused with 4% paraformaldehyde, and the brain, liver, kidney, thymus, spleen, adrenal glands, and knee joints were collected for further analysis.

#### Evaluation of the knee edema

The diameter of both left and right knee joints was measured with an electronic caliper. An increase in knee diameter was used as an indicator of edema caused by the induced inflammatory state.

#### Mechanical allodynia

Mechanical allodynia was evaluated through the flexion and extension test. The animals were submitted to five consecutive flexion and extension movements in both knees. The number of vocalizations during each flexion and extension movement was registered. An increase in the number of vocalizations was used as an indicator of mechanical allodynia development.

#### Mechanical hyperalgesia

The pressure application measurement (PAM) method was employed to evaluate the development of mechanical hyperalgesia. Firstly, the animal was securely held in the experimenter’s arm. A force transducer unit, fitted on the thumb of the experimenter, was placed on one side of the knee joint of the animal, and the forefinger on the other side. A force (range of 0–1500 gf) was gradually applied to the joint until a behavioral response, such as vocalization, limb withdrawal, wriggling, or freezing of whisker movement, was observed. The maximum force applied before the behavioral response was recorded as the Limb Withdrawal Threshold (LWT), measured twice in the ipsilateral and contralateral limbs at 1 min intervals. A decrease in LWT values was used as an indicator of the development of mechanical hyperalgesia.

#### Histology

At the end of the behavioral assessments, the animals were injected with a lethal dose of pentobarbitone intraperitoneally (80 mg/kg) and transcardially perfused with 4% paraformaldehyde. Then, the knee joints, brain, liver, kidney, thymus, spleen, and adrenal glands were immediately immersed in 10% (*v/v*) formalin and stored at RT for at least one week. The knee joint was further dissected to remove the excess tissue and bone, and decalcified with Richard-Allan Scientific decalcifying solution for 2.5 days. Decalcification was confirmed when a needle could be passed through the bone. After, tissue samples were transferred to histological cassettes and processed in a Spin Tissue Processor (STP120-2, Microm, Walldorf, Germany). The processed samples were embedded in paraffin using an Embedding Center (EC350-1/EC350-2, Microm, Walldorf, Germany). Sagittal sections 5 μm thick were cut from the collected tissues using a manual rotary microtome (HM355S, Microm, Walldorf, Germany). The histological slides were placed in an oven at 70 °C for 30 min to melt the paraffin, then deparaffined and rehydrated in an automatic stainer (HMS740, Microm, Walldorf, Germany). Then, the slides were stained with Hematoxylin & Eosin according to a routine protocol using an automatic stainer (HMS740, Microm, Walldorf, Germany). The histological sections of each organ were analyzed under an optical microscope (DM750, Leica, Heerbrugg, Switzerland).

#### Immunohistochemistry analysis

To break the protein crosslinks and, therefore, unmask the antigens and epitopes in formalin-fixed and paraffin-embedded tissue sections, the knee joint histological sections were subjected to heat-induced antigen-retrieval with sodium citrate buffer (10 mM sodium citrate, 0.05% Tween 20, pH 6.0) for 4 min, in a microwave at 200 W. The microwave was stopped every 15 and 15 s in the first 2 min, and then every 5 s to avoid boiling the buffer. The slides were cooled down inside the buffer for approximately 20 min. Then, they were transferred to a StainTray™ black lid staining chamber to ensure a humidified environment and protection from the light. The slides were washed with distilled water three times for 5 min. Membrane permeabilization was achieved using 0.2% Triton X-100 in PBS at RT for 15 min. Afterward, the slides were incubated with 3% BSA freshly prepared in PBS for 30 min at RT to block nonspecific antigen binding. Primary antibodies against IL-6 or CD68, both diluted in 1% BSA in PBS (1:500), were added and incubated overnight at 4 °C. The slides were washed three times with 0.01% Tween-20 in PBS for 5 min each. The secondary antibody Alexa Fluor 594, diluted in 1% BSA in PBS (1:500), was added and incubated for 1 h, at RT, in the dark. Afterward, the histological sections were washed with 0.05% Tween-20 in PBS (3 × 5 min) and then with PBS. Finally, the slides were mounted with Vectashield fluorescence mounting medium and sealed with nail polish. A negative control, constating only of incubation with the secondary antibody, was also prepared to assess for false positives. The histological slides were analyzed under a transmitted and reflected light microscope with an apotome 2 (Axio Imager Z1m, Zeiss, Göttingen, Germany).

### Statistical analysis

Results are expressed as mean ± standard deviation (SD) of 3 independent experiments, with a minimum of 3 replicates for each condition. Statistical analyses were performed using GraphPad Prism 8.0.1 software. Unpaired t-test, one-way or two-way analysis of variance (ANOVA), and Tukey’s multiple comparisons test or Dunnett's multiple comparisons test were used. Differences between experimental groups were considered significant with a confidence interval of 95% whenever *p* < 0.05.

## Results and discussion

As *E. purpurea* traditional preparations are strongly immunomodulatory [55], this study was designed to enhance the therapeutic efficacy of a candidate extract, namely DE-R.

### Chemical characterization of extracts

Table 1 represents the compounds identified by LC-HRMS in DE-R. Four phenols/carboxylic acids and nineteen alkylamides were identified in DE-R. Both product ions and relative intensities of standard fragments (Supplementary Table S1), perfectly matched those obtained for the compounds in DE-R. Supplementary Tables S2 and S3 further show the tR, the precursor ions, and the product ions obtained for phenols/carboxylic acids and alkylamides, respectively. Therefore, DE-R is mainly composed of alkylamides.

**Table 1:** Overview of the identified compounds (phenols/carboxylic acids and alkylamides) in DE-R by LC-HRMS

Chemical compounds	DE -R
Malic Acid	-
Vanillic acid	-
Protocatechuic acid	-
Caftaric acid^a^	-
Chlorogenic acid^a^	-
Quinic acid	-
Vanillin	-
Caffeic acid^a^	-
Benzoic acid	+
Cynarin^a^	-
Echinacoside^a^	-
*p*-coumaric acid	-
Chicoric acid^a^	+
Rutin	+
Quercetin	+
Tetradeca-8Z-ene-11,13-diyn-2-one	-
Pentadeca-8Z-13Z-diene-11-yn-2-one	-
Pentadeca-8Z,11Z,13E/Z-triene-2-one	-
Dodeca-2E,4Z,10E-triene-8-ynoic acid isobutylamide	+
Dodeca-2E,4Z,10Z-triene-8-ynoic acid isobutylamide	+
Dodeca-2E,4E,10Z-triene-8-ynoic acid isobutylamide	+
Dodeca-2Z,4E,10Z-triene-8-ynoic acid isobutylamide	-
Dodeca-2E,4E,10E-triene-8-ynoic acid isobutylamide	+
Undeca-2E,4Z-diene-8,10-diynoic acid isobutylamide	+
Undeca-2E/Z-ene-8,10-diynoic acid isobutylamide^a^	-
Undeca-2Z,4E-diene-8,10-diynoic acid isobutylamide	+
Undeca-2E/Z,4Z/E-diene-8,10-diynoic acid 2-methylbutylamide	-
Pentadeca-2E,9Z-diene-12,14-diynoic acid 2-hydroxyisobutylamide	-
Dodeca-2E,4Z-diene-8,10-diynoic acid isobutylamide	+
Undeca-2E,4E-diene-8,10-diynoic acid isobutylamide	-
Dodeca-2Z,4E-diene-8,10-diynoic acid isobutylamide	+
Dodeca-2E-ene-8,10-diynoic acid isobutylamide^a^	+
Trideca-2E,7Z-diene-10,12-diynoic acid isobutylamide	+
Dodeca-2,4-diene-8,10-diynoic acid 2-methylbutylamide	+
Dodeca-2Z,4Z,10Z-triene-8-ynoic acid isobutylamide	+
Trideca-2E,7Z-diene-10,12-diynoic acid 2-methylbutylamide	+
Dodeca-2E,4E,8Z,10E/Z-tetraenoic acid isobutylamide^a^	+
Dodeca-2E,4Z,10E-triene-8-ynoic acid 2-methylbutylamide *OR* Dodeca-2E-ene-8,10-diynoic acid 2-methylbutylamide	+
Pentadeca-2E,9Z-diene-12,14-diynoic acid isobutylamide	+
Dodeca-2E,4E,8Z-trienoic acid isobutylamide	+
Trideca-2Z,7Z-diene-10,12-diynoic acid 2-methylbutylamide	-
Dodeca-2E,4E,8Z,10E/Z-tetraenoic acid 2-methylbutylamide	+
Hexadeca-2E,9Z-diene-12,14-diynoic acid isobutylamide	-
Dodeca-2E,4E-dienoic acid isobutylamide^a^	+

^a^Injected standards; symbol “ + ” represents the presence of compound; symbol “-” represents the absence of compound

E/Z stereochemistry is indicated here following existing literature [39–45], but it should be highlighted that without NMR spectra, it is not possible to distinguish between E and Z isomers conclusively

### Liposomes characterization

#### PC quantification

Liposomes were used as nanocarriers to encapsulate DE-R, thereby increasing its solubility and therapeutic index. Although an initial lipid concentration of 33 mM was theoretically defined to produce the liposomes, the experimentally determined values were 21.8 ± 2.3 mM for FLPs and 14.1 ± 3.4 mM for FLPs + DE-R. This corresponds to lipid losses of approximately 34% and 57%, respectively. Such reductions are consistent with values reported in the literature, which may be attributed to lipid losses during the preparation and purification steps [56]. The incorporation of alkylamides from DE-R into the lipid bilayer may also affect vesicle formation and recovery.

#### Size distribution and zeta potential

Table 2 represents the size, PDI, and zeta potential of the LPs, FLPs, LPs + DE-R, and FLPs + DE-R. LPs functionalization with folic acid did not affect the size of resulting liposomes (106.3 ± 8.7 nm and 107.0 ± 9.1 nm, respectively). Loading LPs and FLPs with DE-R significantly increased the size of both liposome formulations (113.7 ± 8.7 nm and 114.1 ± 5.1 nm, respectively). As observed for size, the functionalization of liposomes did not affect the PDI of LPs (0.18 ± 0.02 and 0.17 ± 0.03, respectively). The developed liposomal formulations exhibited mean particle size of ≈114 nm, LPs. This size range also facilitates their crossing of the leaky vasculature in inflamed tissues [57, 58]. Consequently, the accumulation of these carriers at the target site can enhance their therapeutic effect and reduce side effects. As M1 macrophages strongly express the folate receptor on their surface, while normal cells display a very low or undetectable expression [32], liposomes were functionalized with folic acid to target these cells actively [59].

**Table 2:** Size, PDI, and zeta potential of LPs, FLPs, LPs + DE-R, and FLPs + DE-R

Formulations	Size (nm)	PDI	Zeta (mV)
LPs	106.3 ± 8.7	0.17 ± 0.03	−3.43 ± 0.41
FLPs	107.0 ± 9.1	0.18 ± 0.02	−2.93 ± 1.54
LPs + DE-R	113.7 ± 8.7 ^a2^	0.12 ± 0.02 ^a3^	−2.46 ± 1.04
FLPs + DE-R	114.1 ± 5.1 ^b1^	0.14 ± 0.02 ^b3^	−3.43 ± 1.67

Statistically significant differences are 1 (*p* < 0.05), 2 (*p* < 0.01), and 3 (*p* < 0.001) in comparison with a (LPs vs. LUVS + DE-R) and b (FLPs vs. FLPs + DE-R)

The loading of LPs and FLPs with DE-R significantly decreased the PDI of both liposome formulations (0.12 ± 0.02 and 0.14 ± 0.02, respectively). Additionally, all the developed formulations were monodispersed in size (PDI < 0.2), indicating homogeneous populations of phospholipid vesicles [60].

The formulations exhibited slightly negative zeta potential values (around −3 mV), but no significant differences were observed in zeta potential with DE-R loading or folic acid functionalization. The zeta potential of LPs was within the −10 to + 10 mV range, indicating neutral samples [61]. The obtained zeta potential values are close to neutrality, which is consistent with the lipid composition of the formulations. The liposomes are primarily composed of EPC, a zwitterionic phospholipid that typically results in near-neutral surface charge [62]. Although colloidal stability is often associated with zeta potential values above ± 25 mV, the incorporation of DSPE-PEG in the formulation provided an alternative stabilization mechanism. PEGylation is well known to enhance colloidal stability by forming a hydrophilic steric barrier around the vesicles, thereby preventing aggregation and improving long-term stability [63].

While increasing liposome size could potentially enhance retention within the synovial cavity, it may also negatively affect the cellular uptake. The mean particle size of our final formulation (≈114 nm) represents a good balance of liposomal stability while maintaining efficient macrophage targeting and internalization, which is critical for maximizing the anti-inflammatory efficacy of the intra-synovial delivery. Nanoparticles with diameters larger than 200 nm are more likely to be internalized via phagocytic mechanisms [64]. Future studies could explore size-tunable formulations to further investigate the trade-off between synovial retention and cellular internalization.

#### Liposomes stability

**Fig. 1:**
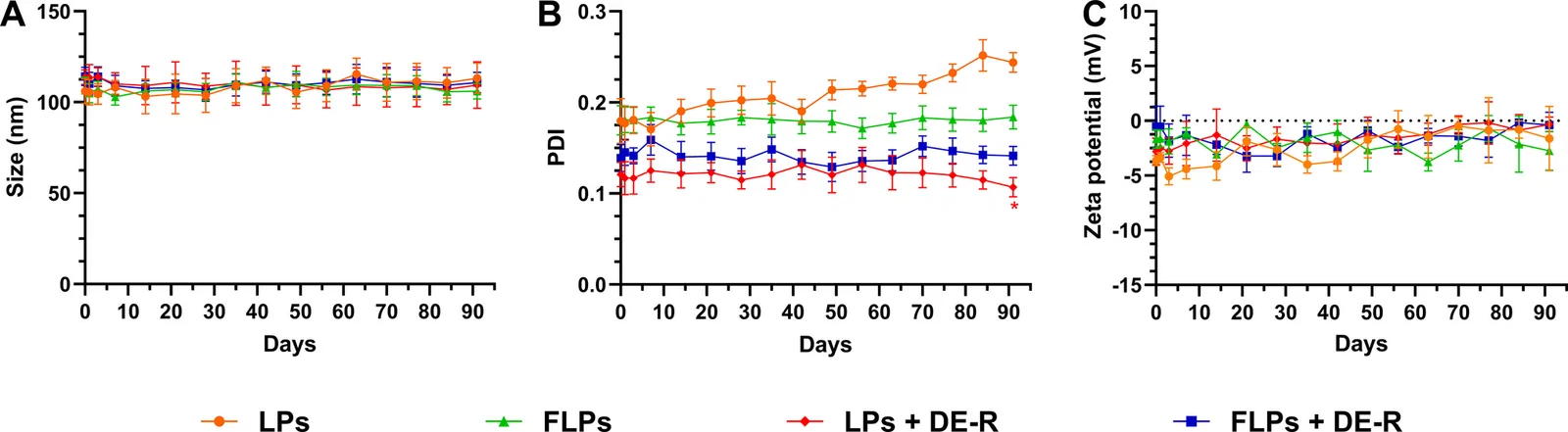
Size (**A**), PDI (**B**), and zeta potential (**C**) of LPs, FLPs, LPs + DE-R, and FLPs + DE-R, suspended in PBS, at 4 °C in the dark, for 90 days

The chemical structure of lipids, which contain functional groups susceptible to oxidation and hydrolysis, makes liposomes prone to rapid degradation [65]. The storage of liposome suspensions in aseptic conditions, at low temperatures (2–8 °C) and protected from light, increases their shelf life [65]. In this work, all the developed formulations were stable for at least 90 days (Fig. 1A), evidencing their capacity to be stored for a considerable period. FLPs, LPs + DE-R, and FLPs + DE-R also showed stable PDI values (Fig. 1B). Only the PDI of LPs increased after approximately 50 days of storage. The zeta potential showed no significant differences over time (Fig. 1C).

#### Entrapment efficiency

As previously referred, DE-R is mainly a mixture of alkylamides, with the isomeric pair dodeca-2E,4E,8Z,10E/Z-tetraenoic acid isobutylamide (alkylamides 8/9) being the most abundant [48]. Hence, this alkylamide was selected to quantify the amount of DE-R entrapped in the liposomes. Importantly, preliminary optimization studies were conducted to assess the influence of DE-R concentration on EE. Two representative concentrations were evaluated: a low concentration (10 mg/mL) and the maximum solubility of the extract in DCM (750 mg/mL). The preliminary results demonstrated that increasing the extract concentration significantly improved EE, reaching approximately 50% under maximum solubility conditions, compared to ≈30% at lower concentrations. Since the highest concentration already showed a clear improvement, testing intermediate values was not considered relevant in the present work.

HPLC analyses demonstrated that 1 mg/mL of DE-R comprises 48.6 ± 7.7 μg/mL of alkylamides 8/9, corresponding to 4.9 ± 0.8% of the whole extract. The DE-R EE, based on alkylamides 8/9 quantification, for LPs + DE-R, was 54.9 ± 19.2%, corresponding to 141.1 ± 35.3 μg/mL of alkylamides 8/9. Because alkylamides are lipophilic, they were incorporated into the lipid bilayer. The DE-R EE decreased with the functionalization with folic acid (26.8 ± 10.2%), being 109.1 ± 9.6 μg/mL of alkylamides 8/9 loaded into the liposome. Indeed, folic acid linked to a saturated lipid can produce a more rigid and stable bilayer structure [66], which makes the entrapment of DE-R more difficult. A similar effect is observed when cholesterol is included liposome formulations [67]. The encapsulation of other plant extracts in liposomes has been previously reported in the literature. Liposomes loading *Lycium barbarum* leaves aqueous ethanolic extracts (EE ≈84%) achieved a mean particle size of 142 nm [68]. Aqueous extracts obtained from *Sambucus ebulus* leaves loaded into EPC:cholesterol liposomes (EE ≈80%) showed a small mean diameter (123 nm), but a highly negative zeta potential (−43 mV) [69]. However, the higher EE observed in these studies may be due to the presence of hydrophilic compounds that can be solubilized in the large aqueous core of LPs. To the best of our knowledge, only one study reported the loading of an *E. purpurea* extract into liposomes (size ≈199 nm and EE ≈80%), but this was obtained using aqueous ethanolic solutions and leaves [70]. However, in that study, no biological evidence was provided. Additionally, we previously demonstrated that DE-R are more effective at reducing an inflammatory response than aqueous and ethanolic extracts from flowers and leaves [25].

### In vitro assays

#### Cytocompatibility of DE-R and liposomal formulations

**Fig. 2:**
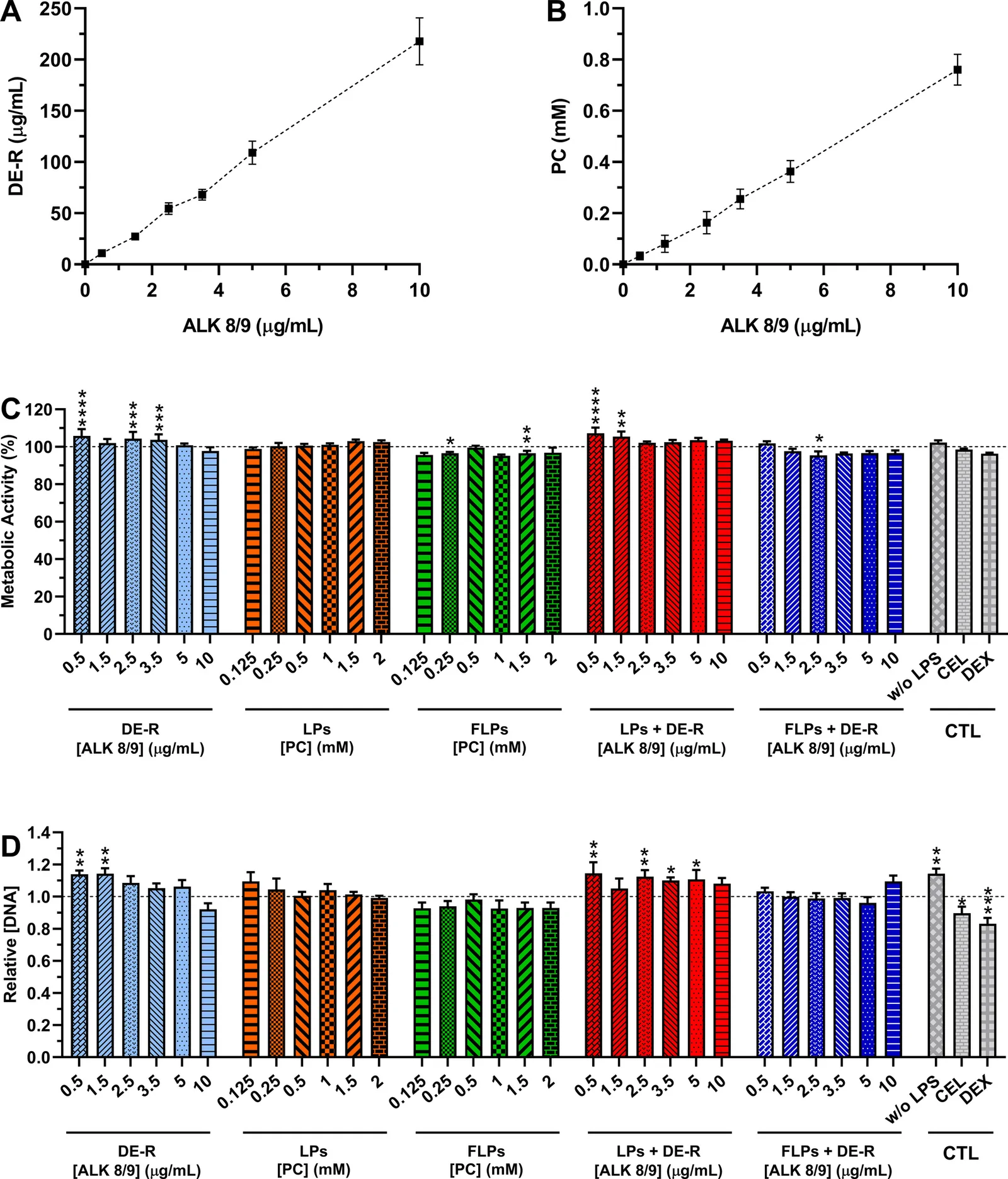
Concentration of alkylamides 8/9 and its correlation with the whole DE-R concentration (**A**) and PC concentration (**B**) used in the biological assays. Metabolic activity (**C**) and relative DNA concentration (**D**) of LPS-stimulated macrophages cultured in the presence of different concentrations of DE-R, LPs, FLPs, LPs + DE-R, FLPs + DE-R, and clinically used anti-inflammatory drugs (celecoxib – CEL – and dexamethasone – DEX, at 10 μM) for 22 h of culture. The dotted line represents the metabolic activity and DNA concentration of the positive control (non-treated LPS-stimulated macrophages, 0 μg/mL). Statistically significant differences are * (*p* < 0.05), ** (*p* < 0.01), *** (*p* < 0.001), and **** (*p* < 0.0001) in comparison to the positive control (non-treated LPS-stimulated macrophages) for each different tested formulation

To demonstrate the efficacy of liposomes as nanocarriers, free DE-R was also evaluated in the biological assays at the same concentrations. As previously mentioned, we established a correlation between the DE-R and alkylamides 8/9 concentrations. Figure 2A shows the relation of the concentration used in the biological assays. The encapsulated alkylamides 8/9 and PC concentrations were also delineated (Fig. 2B). For example, 3.5 μg/mL of alkylamides 8/9 corresponds to 68.1 ± 5.2 μg/mL of whole DE-R and 0.26 ± 0.04 mM of PC. Consequently, the biological effects of the same amount of alkylamides 8/9, added either freely to the cells or loaded into liposomes, can be compared.

The cytocompatibility of DE-R, LPs, FLPs, LPs + DE-R, and FLPs + DE-R was evaluated with LPS-stimulated macrophages, an in vitro model of inflammation [50]. DE-R and all liposomal formulations did not affect the macrophage metabolic activity at any tested concentration compared with control (non-treated LPS-stimulated macrophages, Fig. 2B). Cytocompatibility was also confirmed by the preservation of DNA concentration across all conditions (Fig. 2C). Therefore, the decrease in cytokine levels is only related to the biological activity of the tested formulations.

#### Anti-inflammatory activity

**Fig. 3:**
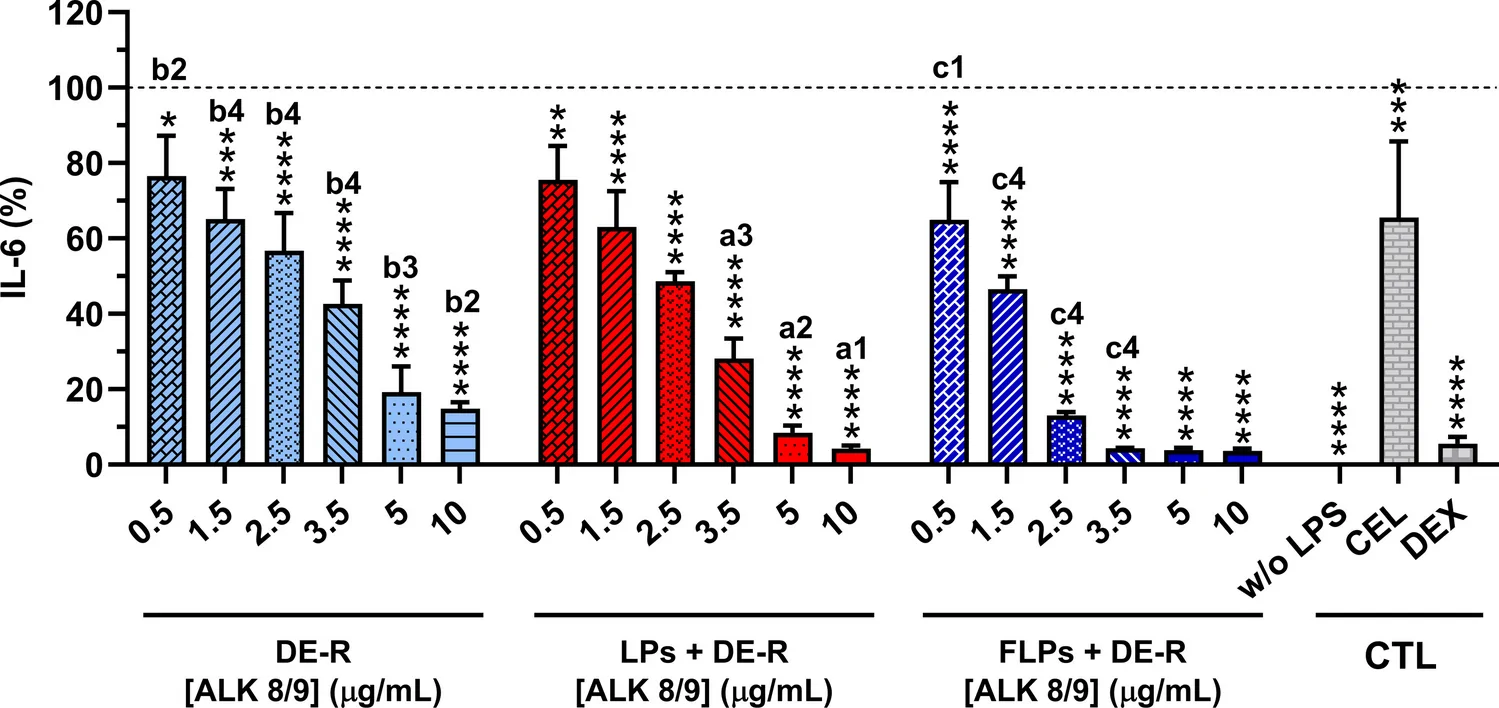
IL-6 (%) produced by LPS-stimulated macrophages cultured in the presence of different concentrations of DE-R, LPs, FLPs, LPs + DE-R, FLPs + DE-R, and clinically used anti-inflammatory drugs (celecoxib – CEL – and dexamethasone – DEX, at 10 μM) for 22 h of culture. The dotted line represents the maximum level of production of the pro-inflammatory cytokine for the positive control (non-treated LPS-stimulated macrophages, 0 μg/mL). Statistically significant differences are* (*p* < 0.05), ** (*p* < 0.01), *** (*p* < 0.001), and **** (*p* < 0.0001) in comparison to the positive control (non-treated LPS-stimulated macrophages) for each different tested formulation, and 1 (*p* < 0.05), 2 (*p* < 0.01), 3 (*p* < 0.001), and 4 (*p* < 0.0001) in comparison with a (DE-R vs. LPs + DE-R), b (DE-R vs. FLPs + DE-R), and c (LPs + DE-R vs. FLPs + DE-R)

The anti-inflammatory activity of the different formulations was evaluated by the decrease of IL-6 concentration in the culture medium. Non-stimulated macrophages did not produce measurable amounts in IL-6 (w/o LPS, Fig. 3). Conversely, the addition of LPS to the macrophages significantly increased the levels of this pro-inflammatory cytokine. Dexamethasone and celecoxib, both at 10 μM, decreased the IL-6 production by 94.4 ± 1.7% and 34.4 ± 20.1%, respectively. As expected, LPs and FLPs demonstrated intrinsic anti-inflammatory activity by reducing the IL-6 levels by 42.2 ± 9.2% and 50.5 ± 7.0%, respectively, at the maximum concentration used in the assays with DE-R (0.76 ± 0.06 mM). In previous work, EPC LPs (120 μM) also decreased the IL-6 production in LPS-stimulated macrophages [46]. LPs and FLPs In the presence of DE-R, LPs + DE-R, and FLPs + DE-R, the IL-6 amount was significantly reduced in a concentration-dependent manner (Fig. 3). DE-R at the highest concentration (10 μg/mL of alkylamides 8/9) strongly reduced the IL-6 production by 85.2 ± 1.7%. A significant increase in the anti-inflammatory effect (1.3-fold) of DE-R was observed when it was encapsulated into LPs at alkylamides 8/9 concentrations of 3.5 μg/mL or higher. Moreover, the entrapped DE-R is in adequate concentration to exert anti-inflammatory effects (109.1 ± 9.6 μg/mL of alkylamides 8/9). These results confirm the high value of liposomes as nanocarriers for drug delivery. Moreover, when folic acid linked to a lipid was incorporated into the liposomal formulation, the effectiveness of the DE-R was significantly enhanced (9.7 times) for concentrations of alkylamides 8/9 higher than 3.5 μg/mL, demonstrating the effectiveness of the active target. LPsLPs Interestingly, the encapsulation of DE-R in FLPs could lead to greater IL-6 reduction at lower DE-R concentrations than with free DE-R. Therefore, the lowest concentration that provides high biological activity was selected for the internalization assay and in vivo experiments (*i.e.,* 3.5 μg/mL of alkylamides 8/9). At this concentration, DE-R decreased IL-6 levels by 57.4 ± 6.2%, and its encapsulation into LPs decreased IL-6 expression by 71.9 ± 5.3%, which was even more efficient when FLPs were used (95.6 ± 0.1% reduction in IL-6). Moreover, FLPs + DE-R demonstrated results similar to or better than those of widely prescribed clinic drugs at comparable concentrations (10 μM of dexamethasone and celecoxib correspond to 3.9 and 3.8 μg/mL, respectively). Dexamethasone was more effective than celecoxib, as expected, due to its different mechanisms of action [71, 72].

#### Liposomes internalization

**Fig. 4:**
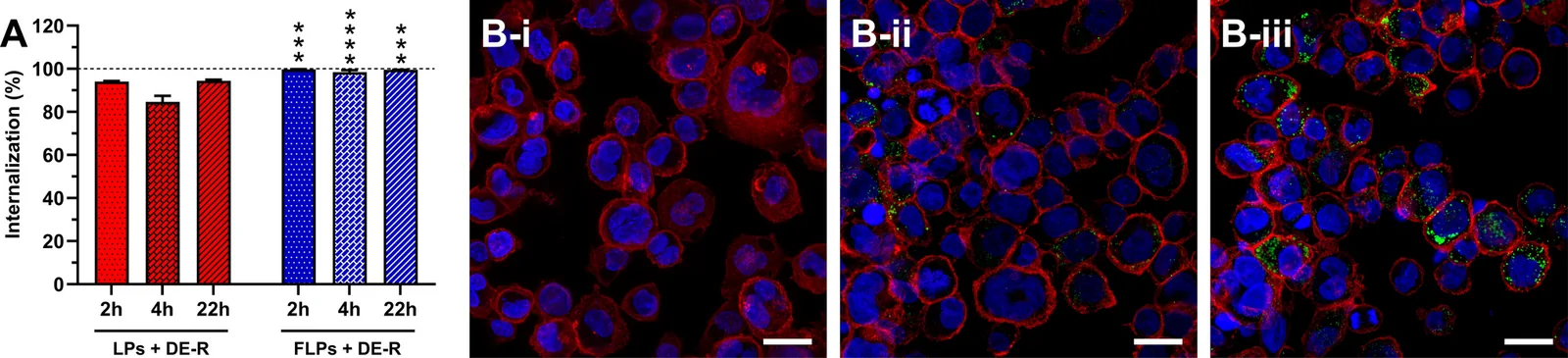
Internalization (%) of LPs + DE-R and FLPs + DE-R after 2, 4, and 22 h of culture with LPS-stimulated macrophages determined with flow cytometry analyses (**A**). Statistically significant differences are *** (*p* < 0.001) and **** (*p* < 0.0001) between LPs + DE-R and FLPs + DE-R. Confocal microscopy images of the non-treated LPS-stimulated macrophages (**B-i**) and LPS-stimulated macrophages cultured with LPs + DE-R (**B-ii**) and FLPs + DE-R (**B-iii**) for 4 h. LPs + DE-R and FLPs + DE-R are highlighted in green (NBD-cholesterol), cell nuclei in blue (DAPI), and the cytoskeleton in red (phalloidin). Scale bar = 25 μM

To exert anti-inflammatory effects, the liposomal formulations must be internalized. The LPs + DE-R and FLPs + DE-R (both containing 3.5 μg/mL of alkylamides 8/9) taken up by LPS-stimulated macrophages were studied by flow cytometry and confocal techniques. Flow cytometry data showed that both formulations are significantly internalized after 2 h of incubation (Fig. 4A). This tendency persisted throughout the experiment. As expected, FLPs + DE-R were more efficiently internalized by LPS-stimulated macrophages (around 99%) than LPs + DE-R (around 91%) in all tested time points, supporting their higher anti-inflammatory activity. Confocal analysis corroborated the high degree of internalization of LPs + DE-R and FLPs + DE-R by this immune cell (Fig. 4B). Those results strongly encouraged us to validate the anti-inflammatory properties of DE-R and FLPs + DE-R in an in vivo model of inflammation.

### In vivo studies

In the second approach of this work, the anti-inflammatory activity of DE-R and FLPs + DE-R was validated in a carrageenan-induced inflammation rat model. The IA administration of carrageenan, a sulfated mucopolysaccharide extracted from seaweeds, induces acute inflammation in the first 24 h and chronic inflammatory hyperalgesia after 1 week [73]. Carrageenan induces synovitis by triggering the accumulation of immune cells and the synthesis and release of inflammatory mediators, leading to edema and pain in the knee joint [74]. Therefore, this in vivo model perfectly mimics an inflammatory scenario and is a good model for studing the effectiveness of new anti-inflammatory formulations. To reduce drug dosage, systemic exposure, and adverse side effects, the formulations were locally administered via IA injection [75].

#### Body weight

The relationship between the extent of joint inflammation and the animals’ weight loss was investigated. Body weight is a crucial parameter indicative of the animals’ healthy when new drugs and formulations are being studied. Indeed, the variations in their body weight could also be correlated with the drug toxicity [76].

During our in vivo experiment, eight rats died just after the IA injections, which can be related to the effect of anesthesia being administered twice in a short time (Sham: *n* = 2; Inflammation control: *n* = 2, Inflammation + FLPs: *n* = 1; Inflammation + DE-R: *n* = 2; Inflammation + celecoxib: *n* = 1). These animals were further excluded from the analysis.

**Fig. 5:**
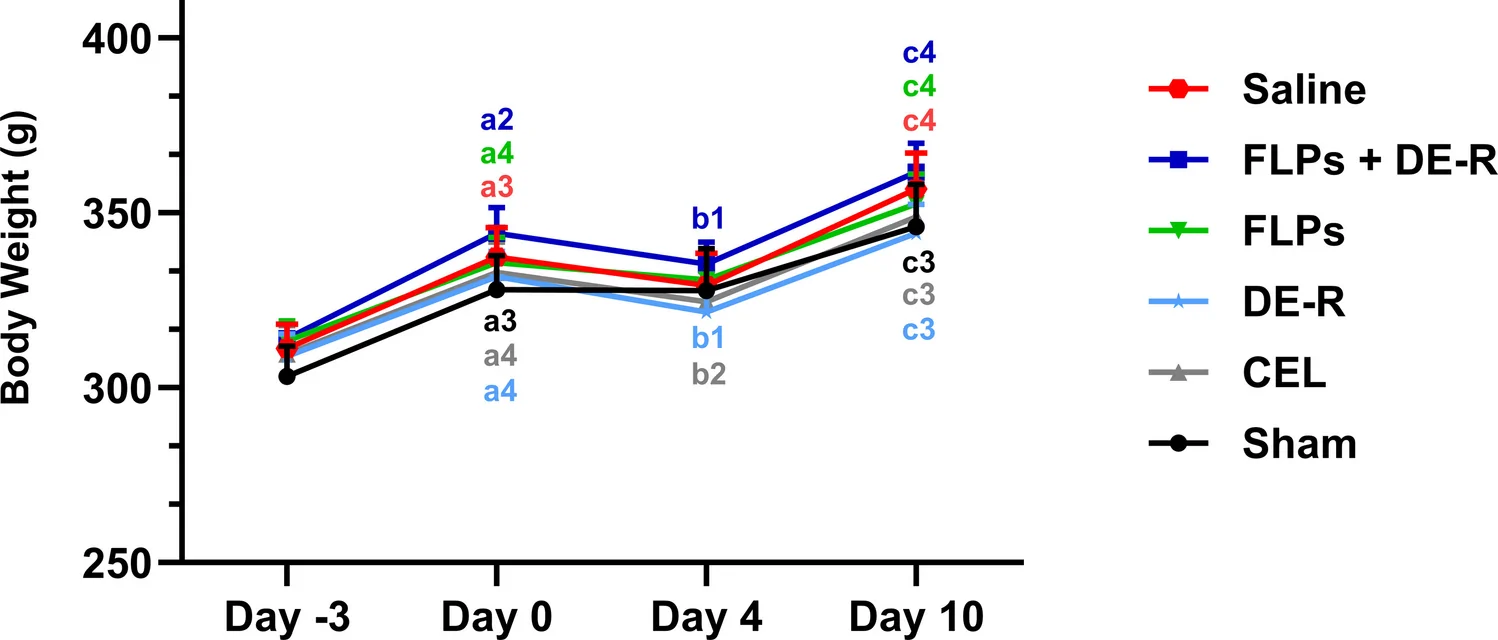
Body weight of non- (Sham) and carrageenan-induced knee inflammation in rats treated with PBS (saline), DE-R (3.5 μg/mL of alkylamides 8/9), FLPs (0.4 mM of PC), FLPs + DE-R (3.5 μg/mL of alkylamides 8/9 and 0.4 mM of PC), and celecoxib (CEL, 10 μM) over the experimental design. Statistically significant differences are 1 (*p* < 0.05), 2 (*p* < 0.01), 3 (*p* < 0.001), and 4 (*p* < 0.0001) between a (Day −3 vs. Day 0), b (Day 0 vs. Day 4), and c (Day 4 vs. Day 10)

The body weight of rats (Fig. 5**)** increased after injection of carrageenan or vehicle until the development of the inflammatory state (day −3 to day 0). Rat body weight significantly decreased after administration of the tested formulations (day 0 to day 4). However, after day 4, body weight gain was observed in all the rats. Figure S2 represents the individual body weight of each rat within the respective group.

#### Edema, mechanical allodynia, and mechanical hyperalgesia

Edema and pain are the major signs of an inflammatory response. Edema is the swelling resulting from the increased permeability of blood vessels and subsequent accumulation of leukocytes in the inflamed tissue [77]. Pain can present with a range of prominent symptoms, including allodynia and hyperalgesia [78]. Allodynia is pain elicited by a stimulus that usually does not cause pain, while hyperalgesia is the increased sensitivity to pain produced by a painful stimulus. Indeed, the responsiveness of peripheral nociceptive neurons is heightened by synovial inflammation, thereby increasing pain sensitivity [12].

**Fig. 6:**
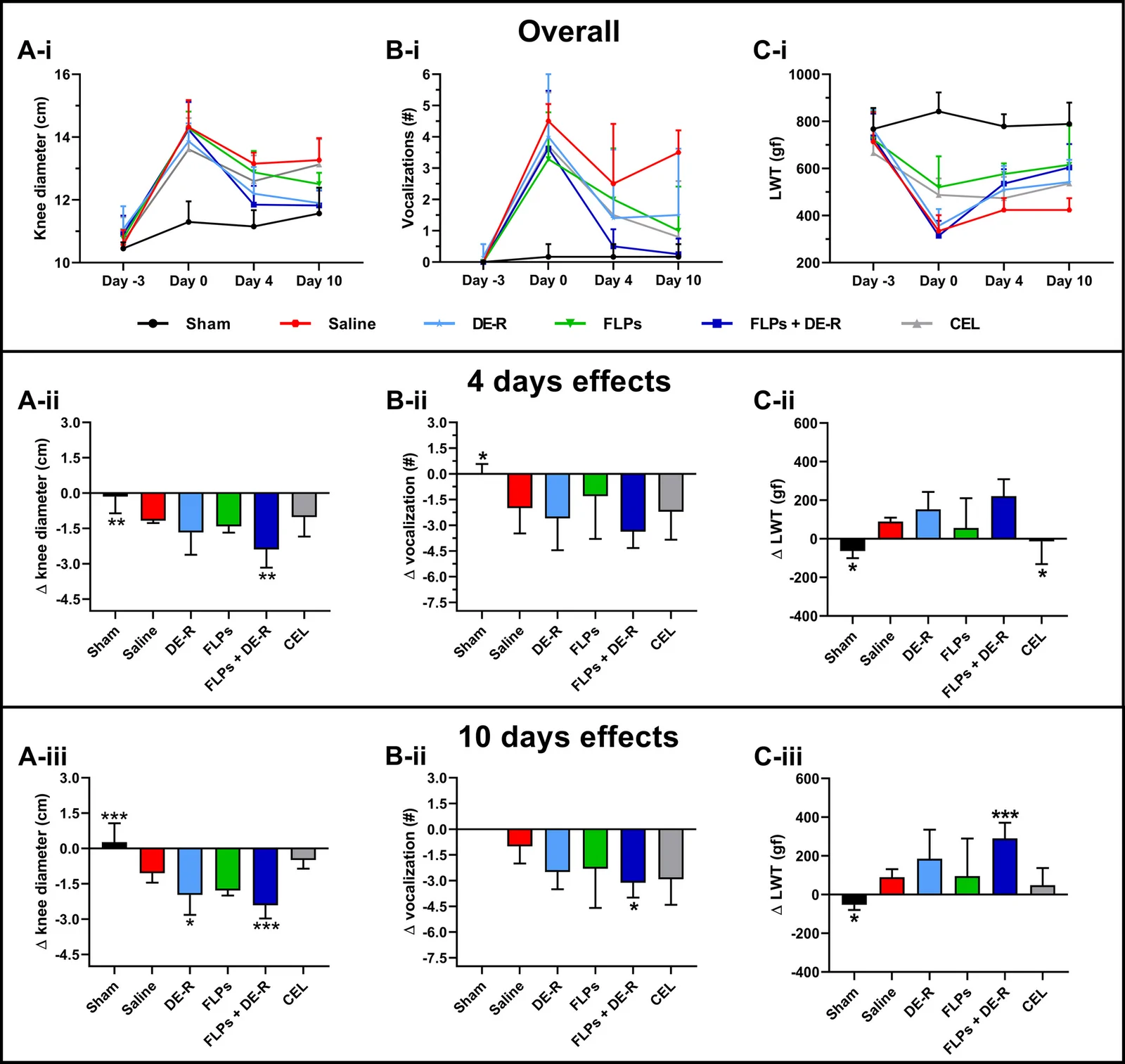
Knee diameter (**A**), number of vocalizations (**B**), and LWT (**C**) of non- (Sham) and carrageenan-induced knee inflammation in rats treated with PBS (saline), DE-R (3.5 μg/mL of alkylamides 8/9), FLPs (0.4 mM of PC), FLPs + DE-R (3.5 μg/mL of alkylamides 8/9 and 0.4 mM of PC), and celecoxib (CEL, 10 μM) over the experimental period. An overall view is presented (**i**), as well as the 4-day effects (difference between day 4 and day 0 for the same group; **ii**), and the 10-day effects (difference between day 10 and day 0 for the same group; **iii**). Statistically significant differences are * (*p* < 0.05), ** (*p* < 0.01), and *** (*p* < 0.001) in comparison with non-treated carrageenan-induced knee inflammation in rats (saline)

The healthy control group injected with PBS (Sham) did not increase knee diameter (Fig. 6A-i), the number of vocalizations (Fig. 6B-i), or LWT (Fig. 6C-i) from day −3 to day 0, as expected. After carrageenan injection, the knee diameter and the number of vocalizations significantly increased. At same time, LWT values decreased in all tested groups, indicating the development of knee inflammation (day −3 to day 0) and mechanical allodynia/hyperalgesia.

After 4 days of treatment, a reduction in knee diameter, from 14.2 ± 0.9 cm to 11.9 ± 0.6 cm, was only observed in the animals treated with FLPs + DE-R, in comparison with the inflammation control group (Fig. 6A-ii). In all treatment groups, the number of vocalizations decreased (Fig. 6B-ii), and, except for celecoxib, LWT increased (Fig. 6C-ii). However, no significant differences were observed in comparison with the inflammation control group.

After 10 days of treatment, animals treated with FLPs + DE-R showed a significant reduction in the diameter of the knees (from 14.2 ± 0.9 cm to 11.8 ± 0.5 cm, Fig. 6A-iii) and the number of vocalizations (from 3.6 ± 1.8 to 0.25 ± 0.5, Fig. 6B-iii), as well as an increase in LWT values (from 314.6 ± 63.2 gf to 604.6 ± 99.1 gf, Fig. 6C-iii). Moreover, 10 days after treatment with FLPs + DE-R, knee diameter and vocalizations were comparable to those of the healthy animals (Fig. 6A-i and B-i). DE-R also significantly reduced knee inflammation (from 13.9 ± 0.6 cm to 11.9 ± 0.4 cm). Treatment with empty FLPs or celecoxib did not significantly affect the amelioration of edema, mechanical allodynia, or mechanical hyperalgesia.

#### Safety

The toxicity of the injected formulations in the main vital organs and the injected knee joint was analyzed by assessing structural and morphological changes in compared with healthy animals (Sham). Hematoxylin–eosin-stained histological analysis of the kidney, liver, spleen, thymus, adrenal gland, cerebellar cortex, cerebrum, and knee joint did not reveal any differences among the experimental groups (Figures S3-S10). Therefore, our formulations did not cause toxic effects in the animals, indicating their safety.

#### Anti-inflammatory activity

**Fig. 7:**
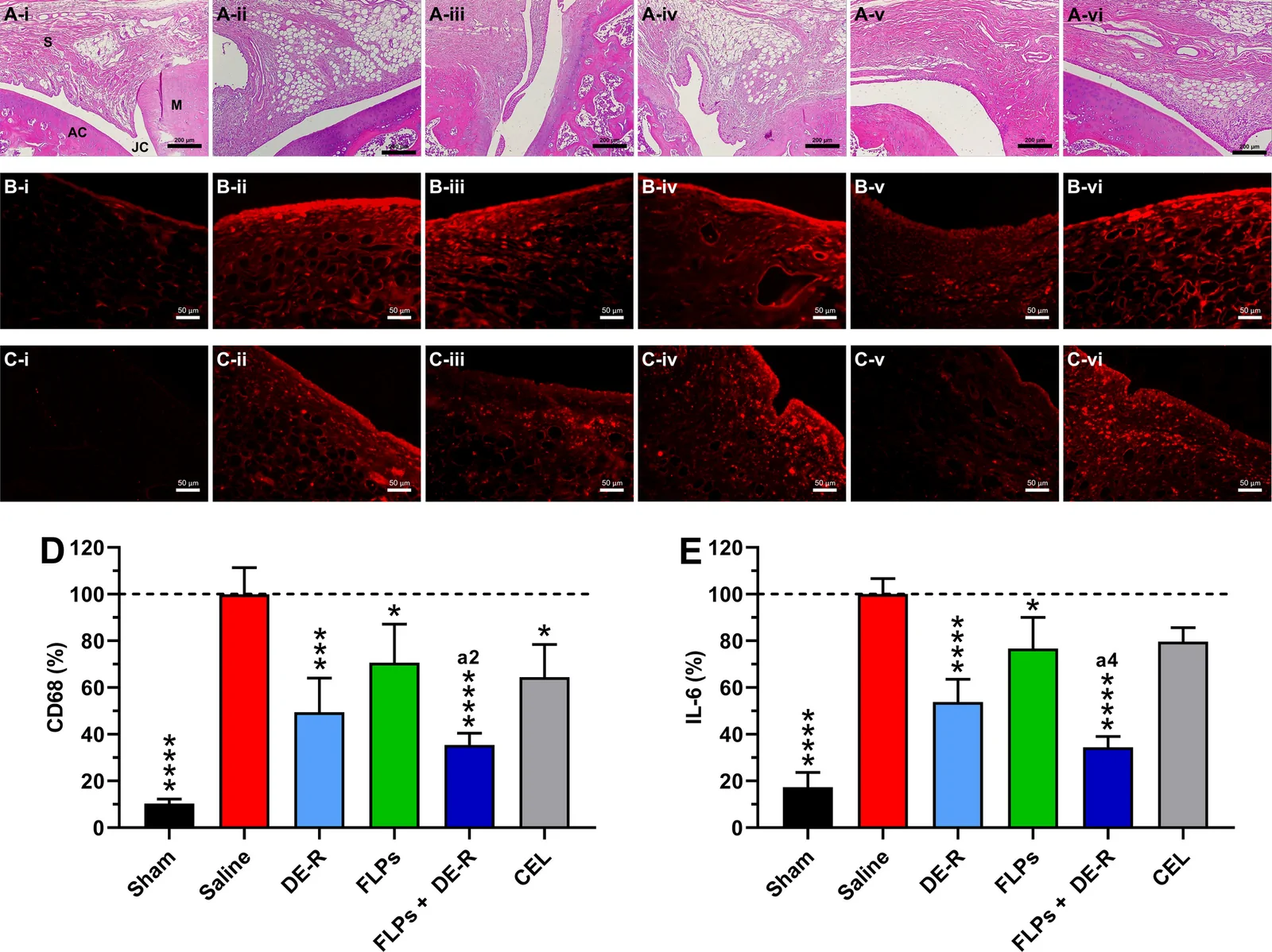
Representative optical images of the histological sections of knee joint stained with hematoxylin and eosin (**A**, 100 × magnification), and immunofluorescence against CD68 (**B**), and IL-6 (**C**) of non- (Sham, **i**), and carrageenan-induced knee inflammation in rats treated with saline (**ii**), DE-R (3.5 μg/mL of alkylamides 8/9, **iii**), FLPs (0.4 mM of PC, **iv**), FLPs + DE-R (3.5 μg/mL of alkylamides 8/9 and 0.4 mM of PC, **v**), and celecoxib (CEL, 10 μM, **vi**) at 10^th^ day. Fluorescence intensity (%) for CD68 expression (**D**) and IL-6 expression (**E**) was measured using ImageJ software. Statistically significant differences are * (*p* < 0.05), *** (*p* < 0.001), and **** (*p* < 0.0001) in comparison to the inflammation control (saline), and 2 (*p* < 0.01), and 4 (*p* < 0.0001) in comparison with a (DE-R vs. FLPs + DE-R). S: synovial membrane; M: meniscus; JC: joint cavity; AC: articular cartilage

Hematoxylin and eosin staining of the knee joint sections was used to evaluate structural and morphological changes in comparison with non- (Sham) and carrageenan-induced knee inflammation in rats (saline). As expected, healthy animals (Sham) showed a normal synovial membrane, containing many fenestrated small blood vessels, with projected folds into the joint cavity (JC) surrounded by the articular cartilage (AC, Fig. 7A-i and Figure S10 A-i). Higher magnification of the fold demonstrated a regular thin intimal layer at the tissue surface contacting the synovial fluid, containing 1 to 4 synoviocytes in depth and a high density of capillaries (Figure S10A-ii). The subintimal layer was composed of prominent regions with dense connective tissue and/or fat. After carrageenan injection, an increase in basophilic staining (purple color), indicating a high infiltration of leukocytes, was observed, supporting the development of an inflammatory state (Fig. 7A-ii and Figure S10B). After 10 days of treatment, the influx of leukocytes into the inflamed synovium was strongly reduced in rats treated with DE-R (Fig. 7A -iii and Figure S10C), and this reduction was even more evident in the FLPs + DE-R condition (Fig. 7A -v and Figure S10E). Both rats treated with FLPs (Fig. 7A -iv and Figure S10D) and with celecoxib (Fig. 7A -vi and Figure S10F) showed slight decreased in immune cell infiltration.

In the synovium, we analyzed CD68 expression, a selective marker of monocytes and macrophages. Similarly, IL-6 fluorescence was used as an indicator of a pro-inflammatory environment. As expected, healthy animals (Sham) presented basal levels of monocytes/macrophages (Fig. 7B-i and D) and IL-6 expression (Fig. 7C-i and E). After carrageenan injection of the, CD68 (Fig. 7B-ii and D) and IL-6 expression intensity (Fig. 7C-ii and E) increased dramatically, supporting the development of an inflammatory state. After 10 days of treatment, DE-R strongly reduced CD68 (Fig. 7B-iii and D) and IL-6 expression intensity (Fig. 7C-iii and E) by 50.5 ± 14.6% and 46.1 ± 9.7%, respectively. Moreover, treatment with FLPs + DE-R significantly reduced both CD68 (Fig. 7B-v and D) and IL-6 (Fig. 7C-v and E) expression intensity by 60.6 ± 5.0% and 65.6 ± 4.6%, respectively FLPs + DE-R. Both rats treated with FLPs (Fig. 7B-iv, 7C-iv, D and E) and celecoxib (Fig. 7B-vi, 7C-vi, D and E) showed slight decrease in CD68 (≈ 33%) and IL-6 (≈ 22%) expression. Therefore, DE-R and FLPs + DE-R efficiently reduced these inflammatory symptoms (Fig. 6) and the key players in the inflamed synovium (Fig. 7). Although celecoxib did not demonstrate significant relief of edema or pain, it reduced the monocyte/macrophage infiltration. Patients may have better responses to these symptoms than those herein reported. In fact, the clinical doses and the duration of celecoxib administration are much higher than the concentrations tested herein [79]. Usually, 200 mg/day is the recommended dosage for oral administration for 12–24 weeks. These results highlight the powerful biological activities of DE-R encapsulated into FLPs as a potent natural anti-inflammatory formulation in the treatment of inflammatory diseases. Moreover, only a few liposomes encapsulating whole plant extracts have been developed for anti-inflammatory purposes [80]. For example, topical application of *Glycyrrhiza glabra* L. root ethanolic extract incorporated in soy PC liposomes (size ≈100 nm, zeta potential ≈−32 mV, EE ≈84%) significantly reduced the edema in TPA-induced cutaneous inflammation in mice [81]. Therefore, our study contributes to validating that the administration of liposomes loaded with anti-inflammatory plant extracts is a viable strategy to control inflammation in vivo.

Although IL-6 was selected as a representative pro-inflammatory marker in the present study, previous reports have demonstrated that DE-R also modulate other key inflammatory mediators, including TNF-α, IL-1β, and ROS/RNS [25, 82]. The present work was designed as a proof-of-concept to demonstrate that liposomal encapsulation preserves and enhances the anti-inflammatory activity of the extract both in vitro and in vivo. Further studies involving a broader panel of inflammatory markers and deeper mechanistic insights will be addressed in future work to better elucidate the underlying pathways.

Our formulation was specifically designed to target activated macrophages and support therapeutic action within the synovial compartment. To further enhance the mean residence time of the developed liposomes in the joint cavity, future strategies could be explored, such as surface modification with biocompatible polymers (e.g., hyaluronic acid) [83], embedded within injectable hydrogel matrices for sustained release [84], or employing additional ligand-targeting approaches [85]. Implementation of these strategies could contribute to improved retention, bioavailability, and enhance the therapeutic efficacy, expanding the potential of these systems for the treatment of joint-related diseases.

## Conclusion

In this work, we successfully developed liposomal formulations functionalized with folic acid for the targeted delivery to pro-inflammatory macrophages, supporting local retention and therapeutic action in the synovial compartment. The DE-R, with strong anti-inflammatory activity, was efficiently encapsulated into the functionalized liposomes. In vitro biological assays showed that these formulations are cytocompatible at concentrations exhibiting anti-inflammatory properties. Moreover, as FLPs strongly enhanced the therapeutic effect of DE-R, showing that a lower concentration of extract could be used. The in vivo study validated the safety and effectiveness of FLPs + DE-R, by reducing edema, pain, and synovitis in a carrageenan-induced inflammation model. Moreover, free DE-R and DE-R-loaded FLPs showed more robust anti-inflammatory activity than conventional NSAIDs widely used in the clinic. Therefore, *E. purpurea* preparations obtained from roots can be used as natural, green, innovative, and powerful new formulations for the treatment of chronic inflammatory diseases.

## Supplementary Information

Below is the link to the electronic supplementary material.

Supplementary file1 (PDF 6042 KB)

## Data Availability

No datasets were generated or analysed during the current study.

## Abbreviations

alkylamides 8/9: Dodeca-2E,4E,8Z,10E/Ztetraenoic acid isobutylamide
bDMARDs: Biologic disease-modifying antirheumatic drugs
BSA: Bovine serum albumin
cDMARDs: Conventional disease-modifying antirheumatic drugs
cRPMI: Complete RPMI (RPMI 1640 medium supplemented with 10% FBS, 1% antibiotic/antimycotic solution, 1% HEPES, 1% glutamax)
DAPI: 4′,6-Diamidino-2-phenylindole
DE: Dichloromethanolic extracts
DE-R: Dichloromethanolic extracts obtained from *Echinacea purpurea* roots
DLS: Dynamic light scattering
DMSO: Dimethyl sulfoxide
DPBS: Dulbecco’s phosphate-buffered saline
DSPE-PEG: 1,2-Distearoyl-sn-glycero-3-phosphoethanolamine-N-[amino(polyethylene glycol)-2000] ammonium salt
DSPE-PEG-Folate: 1,2-Distearoyl-sn-glycero-3-phosphoethanolamine-N-[folate(polyethylene glycol)-2000] ammonium salt
EE: Entrapment efficiency
ELISA: Enzyme-linked immunosorbent assay
EPC: Egg yolk L-α-phosphatidylcholine
EPR: Enhanced permeability and retention
FBS: Fetal bovine serum
FLPs + DE-R: Folic acid-functionalized liposomes loading dichloromethanolic extracts obtained from *Echinacea purpurea* roots
FLPs: Folic acid-functionalized liposomes
FMLVs: Folic acid-functionalized multilamellar liposomes
FMLVs + DE-R: Folic acid-functionalized multilamellar liposomes loading dichloromethanolic extracts obtained from *Echinacea purpurea* roots
HPLC: High performance liquid chromatography
IA: Intra-articular
IL: Interleukin
LC-HRMS: Liquid chromatography-high-resolution mass spectrometry
LPS: Lipopolysaccharides
LPs: Liposomes
LPs + DE-R: Liposomes loading dichloromethanolic extracts obtained from *Echinacea purpurea* roots
LWT: Limb withdrawal threshold
MLVs: Multilamellar vesicles
MLVs + DE-R: Multilamellar vesicles loading dichloromethanolic extracts obtained from *Echinacea purpurea* roots
NBD cholesterol: 22-(N-(7-nitrobenz-2-oxa-1,3-diazol-4-yl)amino)-23,24-bisnor-5-cholen-3βol
NSAIDs: Nonsteroidal anti-inflammatory drugs
PBS: Phosphate-buffered saline
PC: Phosphatidylcholine
PDI: Polydispersity index
PEG: Polyethylene glycol
PMA: Phorbol 12-myristate 13-acetate
RNS: Reactive nitrogen species
ROS: Reactive oxygen species
RPMI: Roswell Park Memorial Institute-1640 medium
RT: Room temperature
TNF: Tumor necrosis factor
