# Supplementary material for: In vivo evaluation of the anti-inflammatory efficacy of liposomes loaded with Echinacea purpurea roots extracts
Source: Drug Deliv Transl Res. 2026 Apr 9;16(11):4346–66. doi: 10.1007/s13346-026-02109-6 (PMC13748030; doi:10.1007/s13346-026-02109-6)
Supplement: Supplementary file 1 [file 13346_2026_2109_MOESM1_ESM.pdf]

Supplementary Material

In vivo evaluation of the anti-inflammatory efficacy of liposomes loaded with *E. purpurea* roots extracts

Sara F. Vieira[1,2], Diana Fonseca-Rodrigues[2,3], Daniel Mendanha[1,2], Vânia I. B. Castro[1,2], Ricardo A. Pires[1,2], Rui L. Reis[1,2], Filipa Pinto-Ribeiro[2,3], Helena Ferreira[1,2*], Nuno M. Neves[1,2*]

[1] 3B's Research Group, I3BS – Research Institute on Biomaterials, Biodegradables and Biomimetics, University of Minho, Headquarters of the European Institute of Excellence on Tissue Engineering and Regenerative Medicine, AvePark, Parque de Ciência e Tecnologia, Zona Industrial da Gandra, 4805-017 Barco, Guimarães, Portugal

[2] ICVS/3B's–PT Government Associate Laboratory, Braga/Guimarães, Portugal

[3] Life and Health Sciences Research Institute (ICVS), School of Health Sciences, University of Minho, Campus de Gualtar, 4710-057 Braga, Portugal

* Corresponding author:

Prof. Nuno M. Neves (nuno@i3bs.uminho.pt) and Dr. Helena Ferreira (helenaferreira@i3bs.uminho.pt), 3B's Research Group, I3Bs – Research Institute on Biomaterials, Biodegradables and Biomimetics, University of Minho, Headquarters of the European Institute of Excellence on Tissue Engineering and Regenerative Medicine, AvePark, Parque de Ciência e Tecnologia, Zona Industrial da Gandra, 4805-017 Barco, Guimarães, Portugal. Tel.: +351-253-510-913 (H.F.); +351-253-510-905 (N.M.N.); Fax: +351-253-510-909 (H.F. & N.M.N.)

Table S1 – LC-HRMS data of caftaric acid, chlorogenic acid, caffeic acid, cynarin, echinacoside, chicoric acid, undeca-2E/Z-ene-8,10-diynoic acid isobutylamide, dodeca-2E-ene-8,10-diynoic acid isobutylamide, dodeca-2E,4E-dienoic acid isobutylamide, and dodeca-2E,4E,8Z,10E/Z-tetraenoic acid isobutylamide.

Standard compound	Chemical structure	Formula	Exact mass	Precursor ion (m/z)	t_R (min)	MS/MS product ions
Caftaric acid	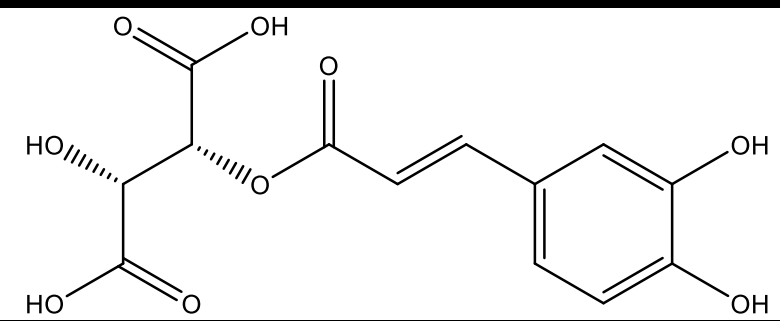	$C_{13}H_{12}O_9$	312.04813196	311.0202 [M-H]-	3.0	135.0372 (100), 179.0241 (31), 149.0004 (17)
Chlorogenic acid	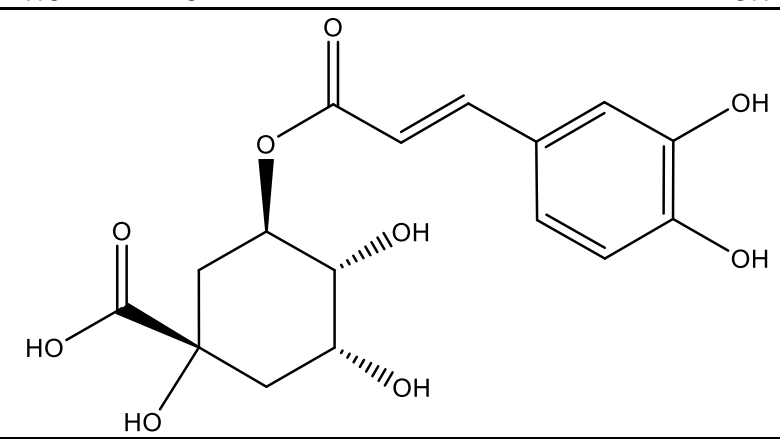	$C_{16}H_{18}O_9$	354.09508215	353.0645 [M-H]-	4.0	191.0443 (100), 192.0479 (6), 161.0147 (2), 85.0247 (2), 127.0328 (1), 135.0373 (1)
Caffeic acid	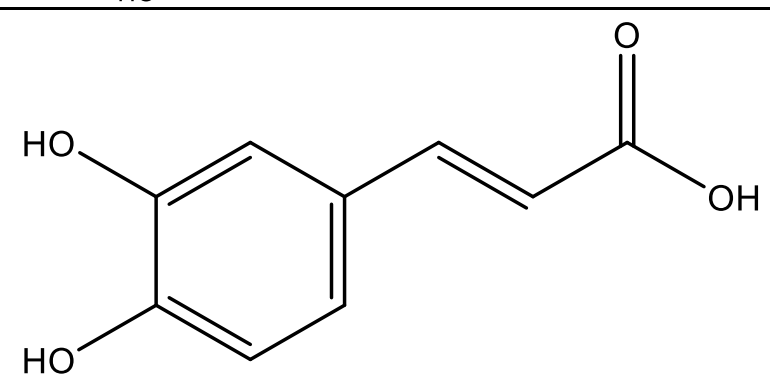	$C_9H_8O_4$	180.04225873	179.0242 [M-H]-	5.1	135.0370 (100), 134.0295 (92), 89.0346 (9), 107.0439 (7), 117.0276 (7), 136.0402 (6)
Cynarin	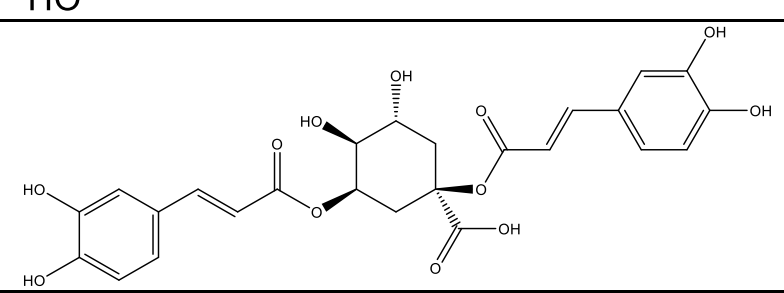	$C_{25}H_{24}O_{12}$	516.12677620	515.0844 [M-H]-	6.3	191.0445 (100), 179.0241 (89), 353.0644 (23), 135.0372 (15), 161.0148 (8), 180.0272 (7), 192.0478 (6), 173.0352 (6), 335.0549 (4), 354.0675 (4)
Echinacoside	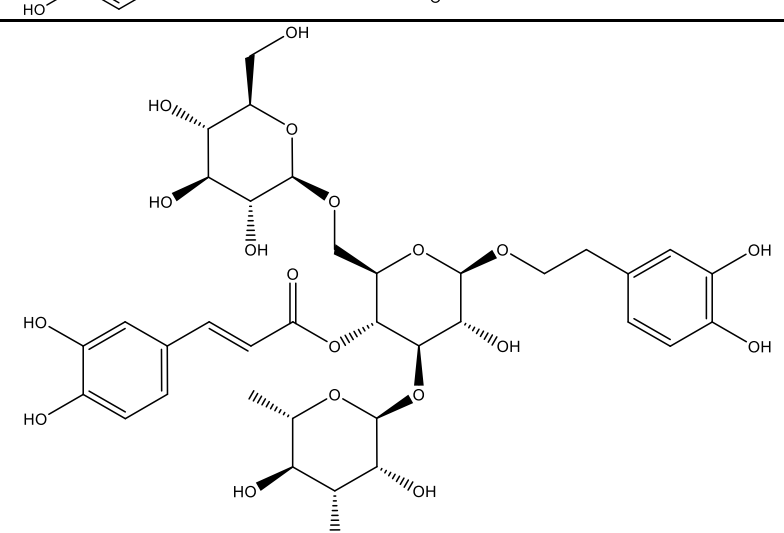	$C_{35}H_{46}O_{20}$	786.25824385	785.1956 [M-H]-	7.4	785.1956 (100), 161.0147 (87), 623.1760 (63), 786.1991 (45), 624.1801 (19), 787.2009 (11), 162.0182 (10), 179.0241 (6)
Chicoric acid	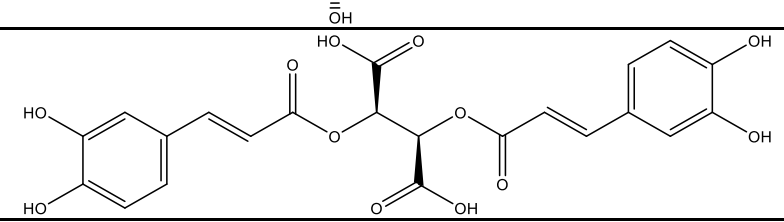	$C_{22}H_{18}O_{12}$	474.07982601	473.0410 [M-H]-	8.9	179.0242 (100), 149.0005 (73), 135.0372 (16), 219.0166 (12), 180.0276 (9), 161.0148 (4), 112.9814 (3), 293.0116 (3)
Undeca-2E/Z-ene-8,10-diynoic acid isobutylamide	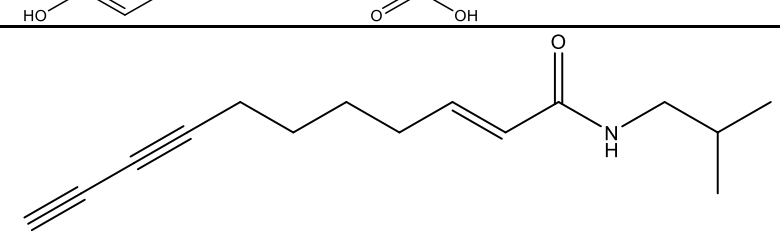	$C_{15}H_{21}NO$	231.16231	232.1677 [M-H]+	20.5	91.0548 (100), 115.0543 (48), 105.0701 (47), 116.0618 (34), 103.0545 (20), 117.0693 (18), 131.0852 (14), 129.0694 (12)

	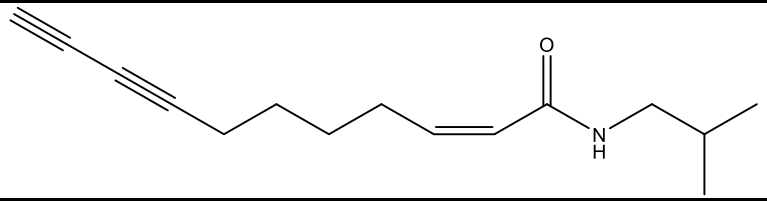					
Dodeca-2E-ene-8,10-diynoic acid isobutylamide	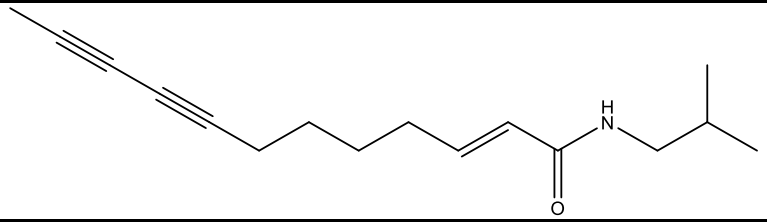	$C_{16}H_{23}NO$	245.17796	246.1831 [M-H]+	20.9	91.0548 (100), 105.0701 (86), 117.0697 (46), 115.0542 (40), 103.0545 (29), 129.0694 (25), 130.0769 (24), 77.0383 (20)
Dodeca-2E,4E,8Z,10E/Z-tetraenoic acid isobutylamide	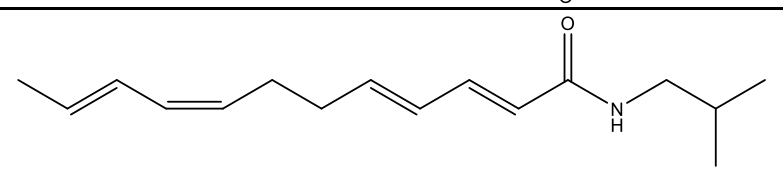 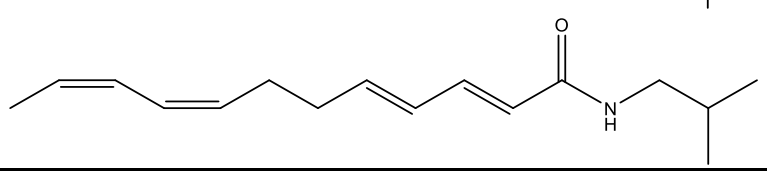	$C_{16}H_{25}NO$	247.19361	248.1986 [M-H]+	21.2	152.1062 (100), 95.0496 (58), 96.0450 (57), 110.0601 (17), 124.0755 (14), 167.1292 (12), 153.1096 (10), 105.0701 (10), 91.0547 (10), 112.0756 (9)
Dodeca-2E,4E-dienoic acid isobutylamide	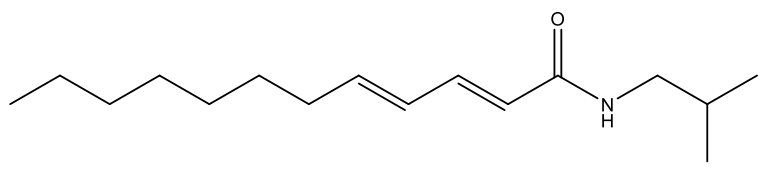	$C_{16}H_{29}NO$	251.22491	252.2307 [M-H]+	22.0	95.0860 (100), 98.0604 (91), 95.0496 (52), 81.0696 (44), 81.0331 (40), 196.1680 (33), 112.0757 (27), 109.1013 (27), 126.0911 (25)

Table S2 – Phenolic/carboxylic acid compounds tentatively identified in DE-R by LC-HRMS (negative mode).

Proposed acid or phenolic compound	Precursor ion [M – H]⁻ (m/z)	t_R (min)	Product ions (m/z) [1]
Benzoic acid	121.0234	6.0	92.0223 (100), 93.0261 (14), 121.0225 (11), 120.0151 (10)
Chicoric acid[a]	473.0427	8.9	179.0253 (100), 149.0016 (69), 135.0383 (14), 219.0176 (10), 180.0287 (10), 129.9692 (5), 112.9822 (4), 161.0164 (4)
Rutin	609.1418	12.7	301.0535 (100), 146.9586 (64), 129.9694 (47), 299.0388 (22), 302.0570 (21), 300.0411 (9), 609.1445 (3)
Quercetin	301.0357	18.4	184.0060 (21), 299.1353 (6), 121.0236 (1), 301.1845 (1), 120.9396 (1), 133.0066 (1), 175.0327 (1), 300.1378 (1), 179.1136 (1), 209.1029 (1), 151.0566 (1)

[a] Injected standards

[1] The MS^2 data were obtained from the fragmentation of the $[M – H]^-$ precursor ion of phenolic compounds. Relative intensities of product ions are in parentheses.

Table S3 – Alkylamides compounds tentatively identified in DE-R by LC-HRMS (positive mode).

Proposed alkylamide compound	Precursor ion $[M + H]^+$ (m/z)	t_R (min)	Product ions (m/z) [1]
Dodeca-2E,4Z,10E-triene-8-ynoic acid isobutylamide	246.1935	14.9	119.0858 (97), 145.1010 (69), 93.0706 (47), 128.1070 (39), 173.0957 (9), 154.1220 (2), 190.1231 (1)
Dodeca-2E,4Z,10Z-triene-8-ynoic acid isobutylamide	246.1935	15.5	119.0857 (75), 145.1008 (53), 93.0709 (34), 128.1072 (24), 173.0954 (9), 190.1221 (2), 154.1220 (2)
Dodeca-2E,4E,10Z-triene-8-ynoic acid isobutylamide	246.1832	19.0	145.1009 (100), 128.0619 (49), 119.0857 (14), 93.0706 (4), 190.1215 (2), 154.1224 (2), 173.0954 (1), 102.0462 (1)
Dodeca-2E,4E,10E-triene-8-ynoic acid isobutylamide	246.1832	20.1	128.0624 (77), 119.0857 (34), 93.0705 (21), 145.065 (18), 173.5704 (6), 154.0636 (5), 190.1219 (4)
Undeca-2E,4Z-diene-8,10-diynoic acid isobutylamide	230.1526	20.3	128.0622 (100), 91.0551 (51), 115.0546 (47), 152.1067 (41), 116.0619 (27), 129.0686 (23), 103.0548 (9), 117.0661 (3), 102.0469 (2), 157.0643 (1), 174.0910 (1), 142.0415 (0.1), 188.1424 (0.1), 202.1199 (0), 57.0694 (0)
Undeca-2Z,4E-diene-8,10-diynoic acid isobutylamide	230.1542	20.6	128.0621 (100), 91.0551 (41), 115.0546 (35), 129.0687 (25), 116.0620 (24), 103.0548 (8), 117.0598 (5), 157.0644 (2), 102.0470 (2), 174.0905 (1), 188.1422 (0.4), 142.0639 (0.2), 202.1213 (0.1), 74.0148 (0), 57.0696 (0)
Dodeca-2E,4Z-diene-8,10-diynoic acid isobutylamide	244.1698	20.7	128.0622 (100), 91.0551 (24), 105.0704 (17), 117.0700 (16), 143.0851 (9), 142.0765 (3), 171.0795 (2), 154.0654 (2), 188.1060 (2), 102.0470 (1), 216.1369 (0.1), 202.1573 (0.1), 74.0147 (0), 57.0695 (0)
Dodeca-2E-ene-8,10-diynoic acid isobutylamide[a]	246.1834	20.9	128.0622 (100), 91.0552 (53), 105.0705 (50), 115.0547 (49), 117.0701 (38), 129.0688 (30), 130.0771 (20), 103.0549 (18), 77.0387 (6)
Dodeca-2Z,4E-diene-8,10-diynoic acid isobutylamide	244.1684	21.0	128.0622 (100), 91.0551 (35), 105.0705 (23), 117.0700 (18), 143.0852 (7), 142.0765 (2), 154.0658 (2), 171.0799 (2), 102.0471 (2), 188.106 (1), 202.1580 (0.2), 216.1366 (0.2), 74.0152 (0)
Dodeca-2,4-diene-8,10-diynoic acid 2-methylbutylamide	258.1846	21.1	91.0552 (24), 117.0702 (24), 105.0705 (22), 143.0853 (13), 116.0607 (7), 142.0767 (4), 171.0798 (3), 188.1061 (2, 168.0803 (2), 156.0613 (1), 202.1214 (0.1), 230.1888 (0.1), 88.1120 (0.1), 71.0859 (0)
Trideca-2E,7Z-diene-10,12-diynoic acid isobutylamide	258.1846	21.1	128.0623 (100), 117.0702 (24), 103.0549 (8), 142.0767 (4), 131.0793 (2), 156.0613 (1), 102.0471 (1), 157.0642 (0.4), 185.0834 (0.1), 202.1214 (0.1), 74.0152 (0)
Dodeca-2Z,4Z,10Z-triene-8-ynoic acid isobutylamide	246.1834	21.2	128.0622 (12), 145.1010 (7), 119.0859 (7), 93.0707 (6), 102.0919 (3), 173.0953 (1), 154.1180 (0.4), 190.1219 (0.2), 57.0695 (0.1), 74.0167 (0)
Trideca-2E,7Z-diene-10,12-diynoic acid 2-methylbutylamide	272.1987	21.2	116.0608 (18), 157.1003 (4), 202.1210 (2), 185.0947 (1), 88.1121 (0.3)

Dodeca-2E,4Z,10E-triene-8-ynoic acid 2-methylbutylamide OR Dodeca-2E-ene-8,10-diynoic acid 2-methylbutylamide	260.1983	21.3	145.1009 (30), 119.0859 (23), 116.0615 (17), 142.0771 (5), 173.0953 (4), 190.1221 (2), 88.1122 (0.3)
Dodeca-2E,4E,8Z,10E/Z-tetraenoic acid isobutylamide[a]	248.2023	21.3	152.1068 (100), 95.0500 (56), 96.0454 (56), 110.0606 (16), 124.0760 (13), 167.1297 (12), 91.0552 (11), 153.1101 (10), 105.0705 (10), 112.0761 (10)
Pentadeca-2E,9Z-diene-12,14-diynoic acid isobutylamide	286.2141	21.5	102.0921 (3), 185.1321 (1), 230.1527 (0.4), 213.1217 (0.1)
Dodeca-2E,4E,8Z,10E/Z-tetraenoic acid 2-methylbutylamide	262.2155	21.5	116.1073 (3), 147.1166 (2), 175.111 (1), 88.1120 (1), 192.1375 (0.4), 71.0854 (0.1)
Dodeca-2E,4E,8Z-trienoic acid isobutylamide	250.2147	21.6	97.0498 (3), 102.0920 (3), 168.1339 (2), 154.1207 (2), 83.0367 (2), 194.1530 (1), 149.1321 (1), 177.1265 (0.4), 57.0694 (0.1)
Dodeca-2E,4E-dienoic acid isobutylamide[a]	252.2306	22.0	95.0864 (100), 98.0609 (90), 95.0500 (52), 81.0699 (44), 81.0335 (41), 196.1687 (32), 112.0762 (28), 109.1018 (28), 128.1436 (25), 126.0917 (24)

[a] Injected standards

[1] The MS^2 data were obtained from the fragmentation of the $[M + H]^+$ precursor ion of alkylamides. Relative intensities of product ions are in parentheses.

E/Z stereochemistry is indicated here following the literature [1–7], but it should be highlighted that without NMR spectra, it is not possible to conclusively distinguish between E and Z isomers.

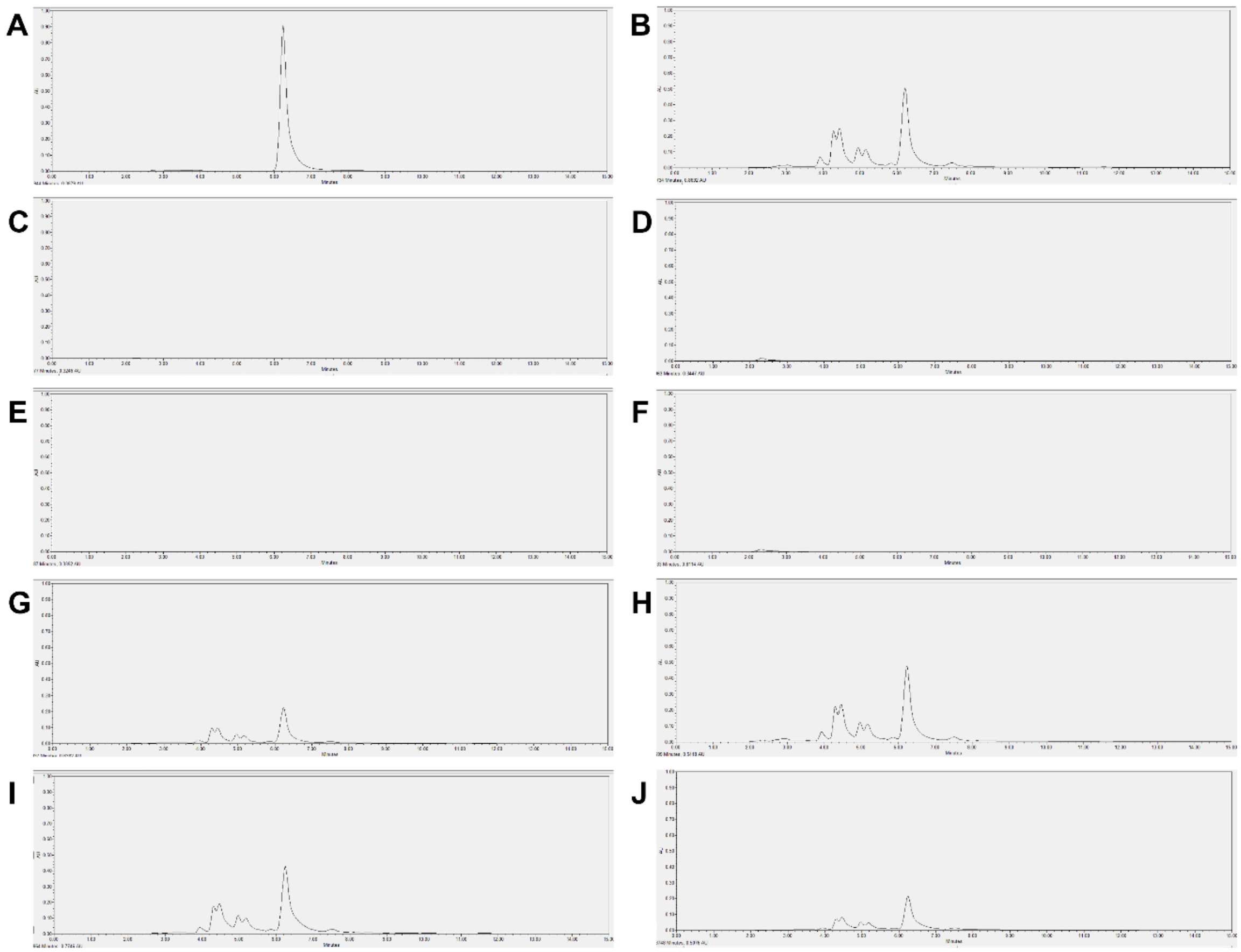

Figure S1 – Analytical HPLC chromatograms of alkylamides 8/9 standard at 100 µg/mL (**A**), DE-R at 1 mg/mL (**B**), MLVs (**C,** 1:4), FMLVs (**D,** 1:4), LPs (**E,** 1:4), FLPs (**F,** 1:4), MLVs + DE-R (**G**, 1:10), FMLVs + DE-R (H, 1:10), LPs + DE-R (I, 1:4), and FLPs + DE-R (J, 1:4); using an UV detection at 254 nm and a flow rate of 1 mL/min.

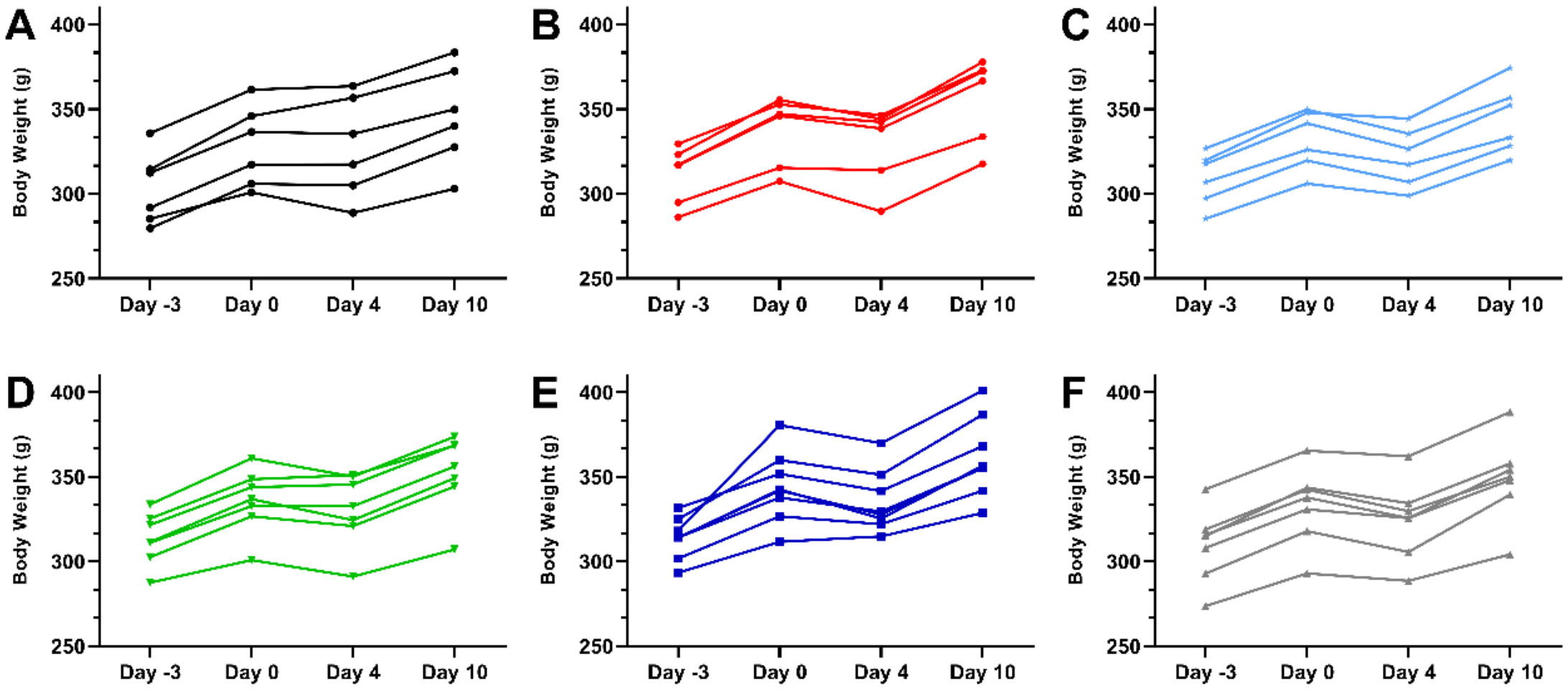

Figure S2 – Body weight of individual non- (Sham, **A**) and carrageenan-induced knee inflammation in rats treated with PBS (saline, **B**), DE-R (3.5 µg/mL of alkylamides 8/9, **C**), FLPs (0.4 mM of PC, **D**), FLPs + DE-R (3.5 µg/mL of alkylamides 8/9 and 0.4 mM of PC**, E**), and celecoxib (10 µM, **F**) over the experimental timeframe.

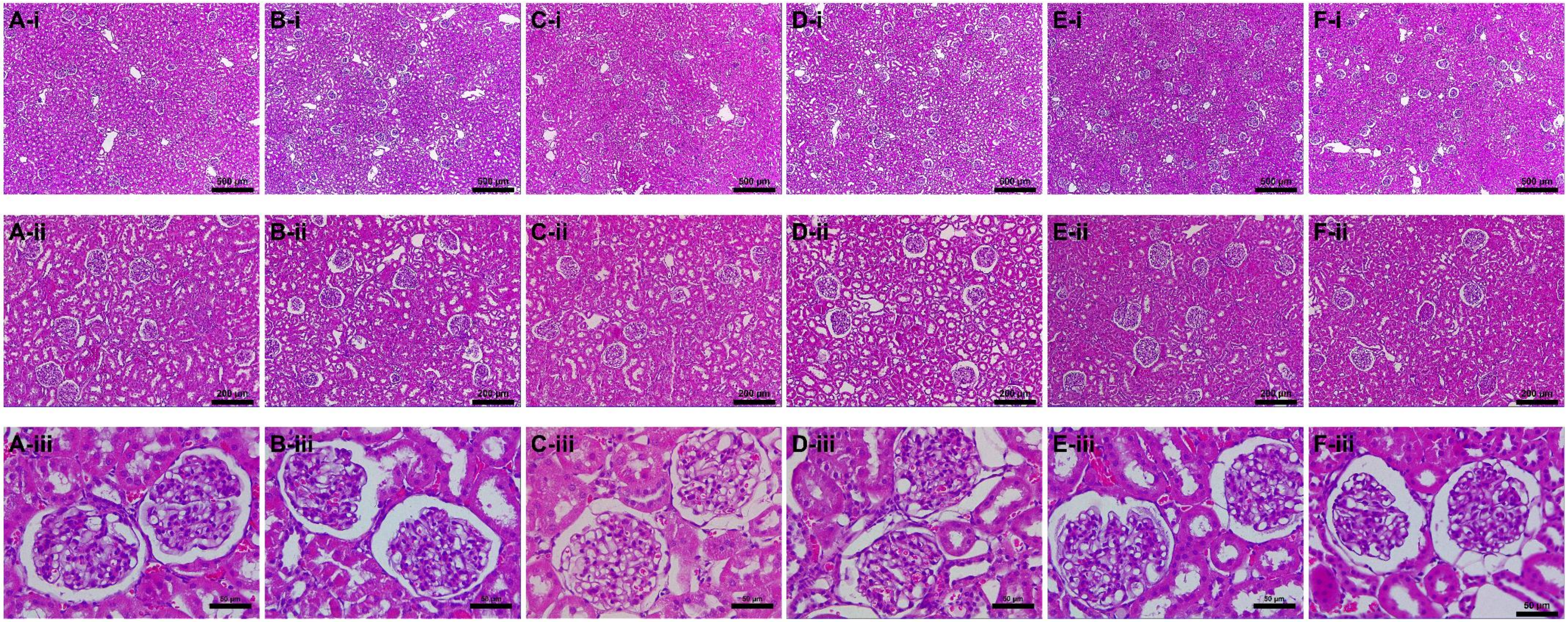

Figure S3 – Representative optical images of the histological sections of the kidney, at 40x (**i**), 100x (**ii**), and 400x magnification (**iii**) stained with hematoxylin-eosin, of non- (Sham, **A**) and carrageenan-induced knee inflammation in rats treated with PBS (saline, **B**), DE-R (3.5 µg/mL of alkylamides 8/9, **C**), FLPs (0.4 mM of PC, **D**), FLPs + DE-R (3.5 µg/mL of alkylamides 8/9 and 0.4 mM of PC, **E**), and celecoxib (10 µM, **F**) at 10th day.

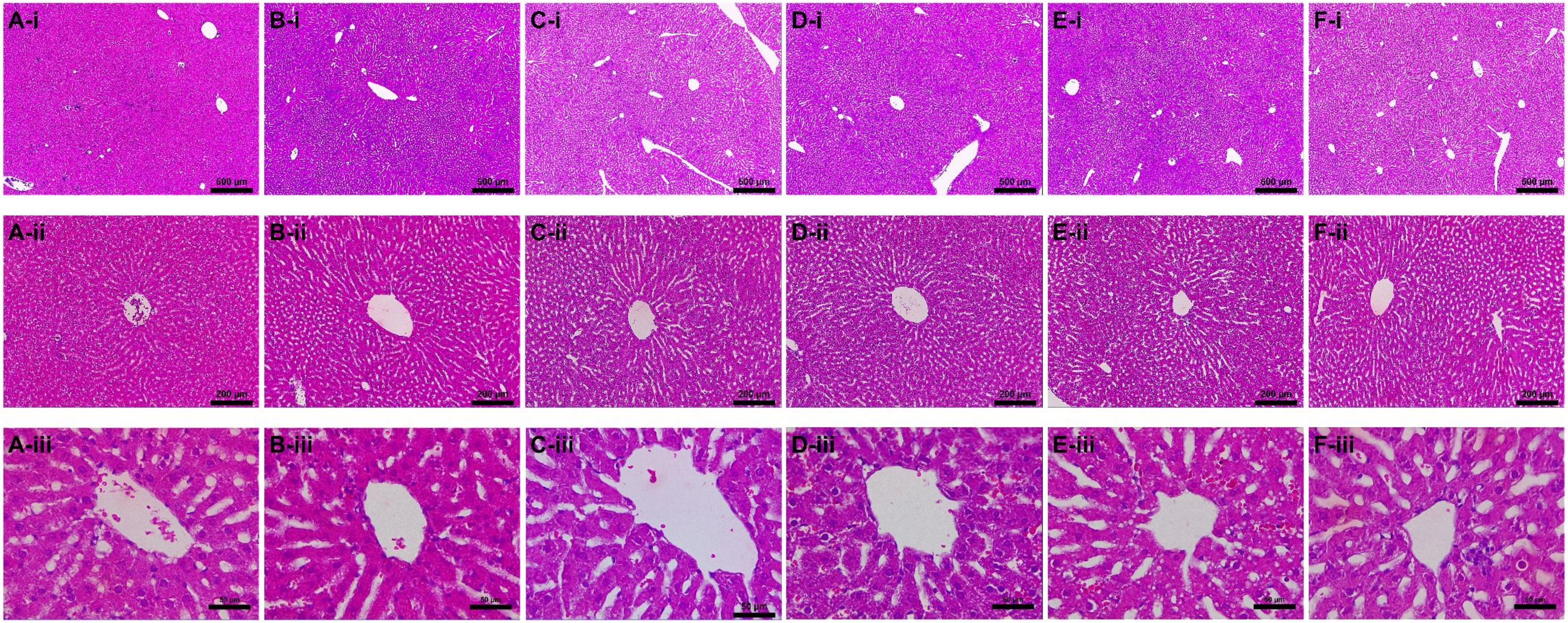

Figure S4 – Representative optical images of the histological sections of the liver, at 40x (**i**), 100x (**ii**), and 400x magnification (**iii**) stained with hematoxylin-eosin, of non- (Sham, **A**) and carrageenan-induced knee inflammation in rats treated with PBS (saline, **B**), DE-R (3.5 μg/mL of alkylamides 8/9, **C**), FLPs (0.4 mM of PC, **D**), FLPs + DE-R (3.5 μg/mL of alkylamides 8/9 and 0.4 mM of PC, **E**), and celecoxib (10 μM, **F**) at 10th day.

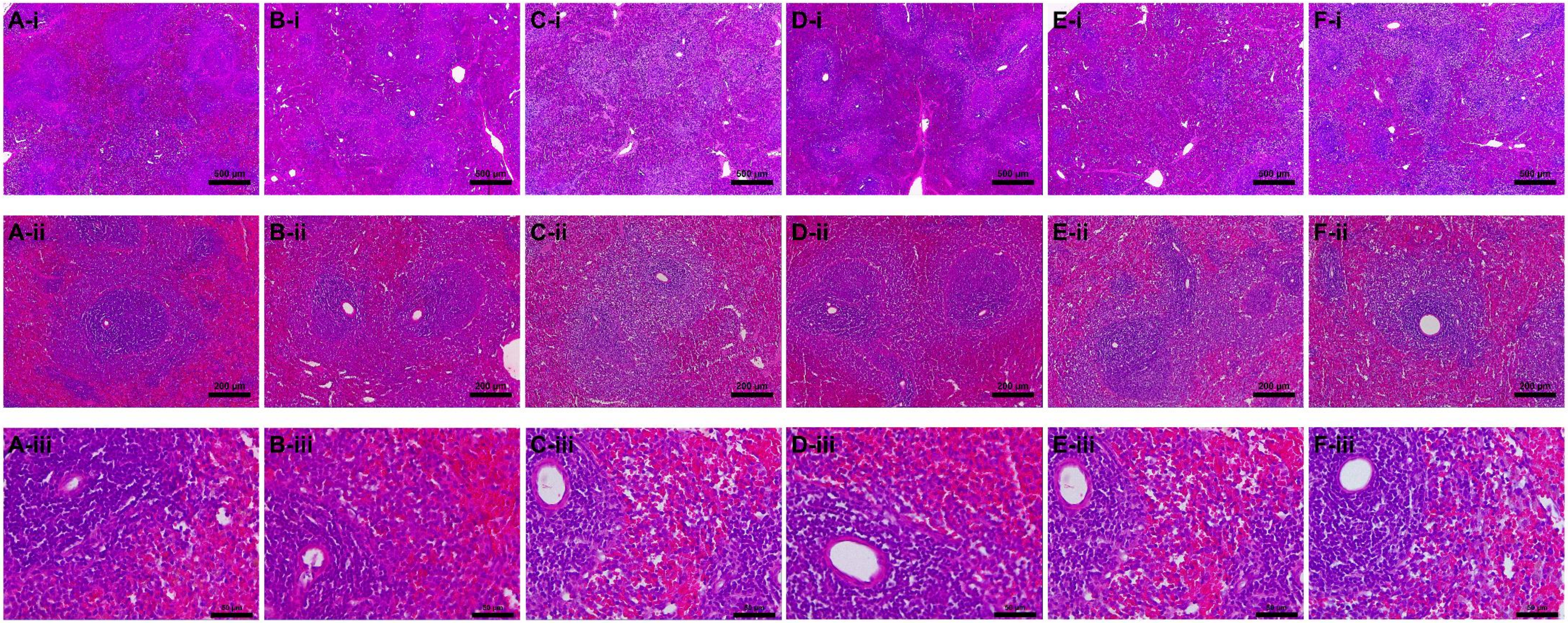

Figure S5 – Representative optical images of the histological sections of the spleen, at 40x (**i**), 100x (**ii**), and 400x magnification (**iii**) stained with hematoxylin-eosin, of non- (Sham, **A**) and carrageenan-induced knee inflammation in rats treated with PBS (saline, **B**), DE-R (3.5 µg/mL of alkylamides 8/9, **C**), FLPs (0.4 mM of PC, **D**), FLPs + DE-R (3.5 µg/mL of alkylamides 8/9 and 0.4 mM of PC, **E**), and celecoxib (10 µM, **F**) at 10th day.

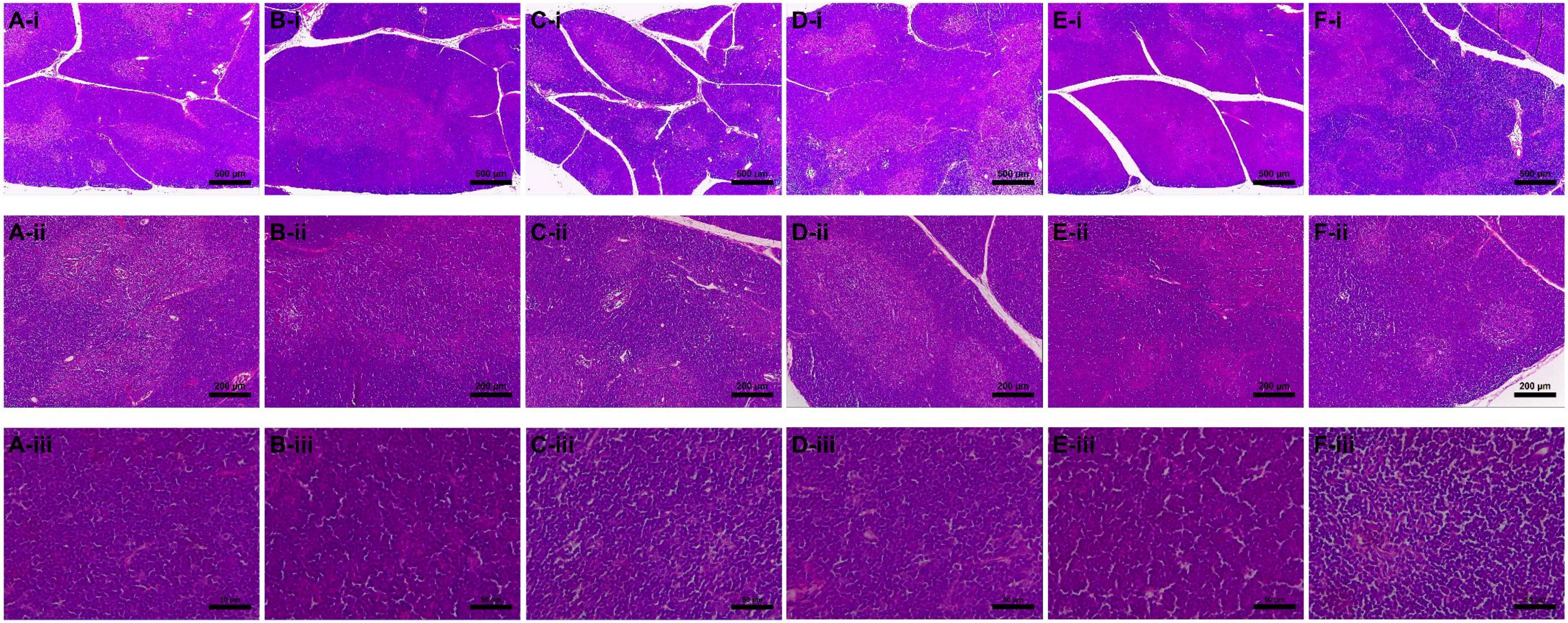

Figure S6 – Representative optical images of the histological sections of the thymus, at 40x (**i**), 100x (**ii**), and 400x magnification (**iii**) stained with hematoxylin-eosin, of non- (Sham, **A**) and carrageenan-induced knee inflammation in rats treated with PBS (saline, **B**), DE-R (3.5 µg/mL of alkylamides 8/9, **C**), FLPs (0.4 mM of PC, **D**), FLPs + DE-R (3.5 µg/mL of alkylamides 8/9 and 0.4 mM of PC, **E**), and celecoxib (10 µM, **F**) at 10th day.

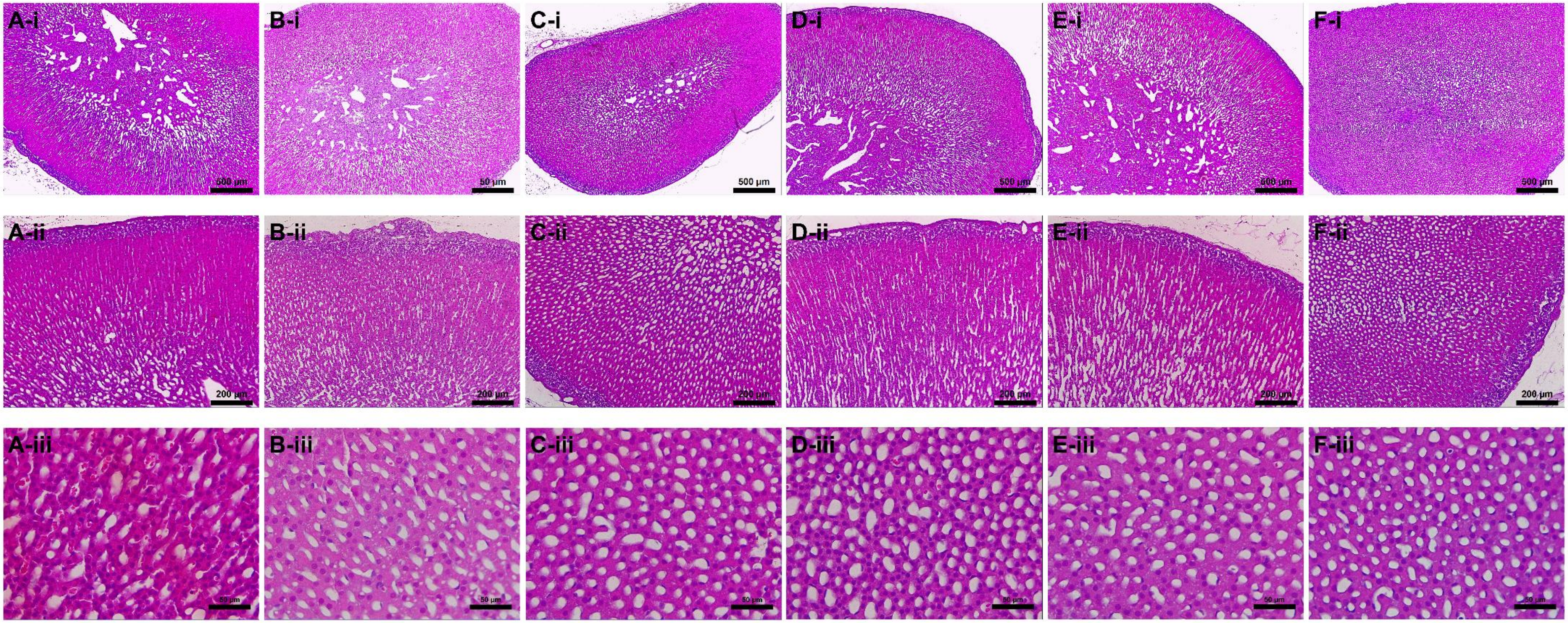

Figure S7 – Representative optical images of the histological sections of the adrenal gland, at 40x (**i**), 100x (**ii**), and 400x magnification (**iii**) stained with hematoxylin-eosin, of non-(Sham, **A**) and carrageenan-induced knee inflammation in rats treated with PBS (saline, **B**), DE-R (3.5 µg/mL of alkylamides 8/9, **C**), FLPs (0.4 mM of PC, **D**), FLPs + DE-R (3.5 µg/mL of alkylamides 8/9 and 0.4 mM of PC, **E**), and celecoxib (10 µM, **F**) at 10th day.

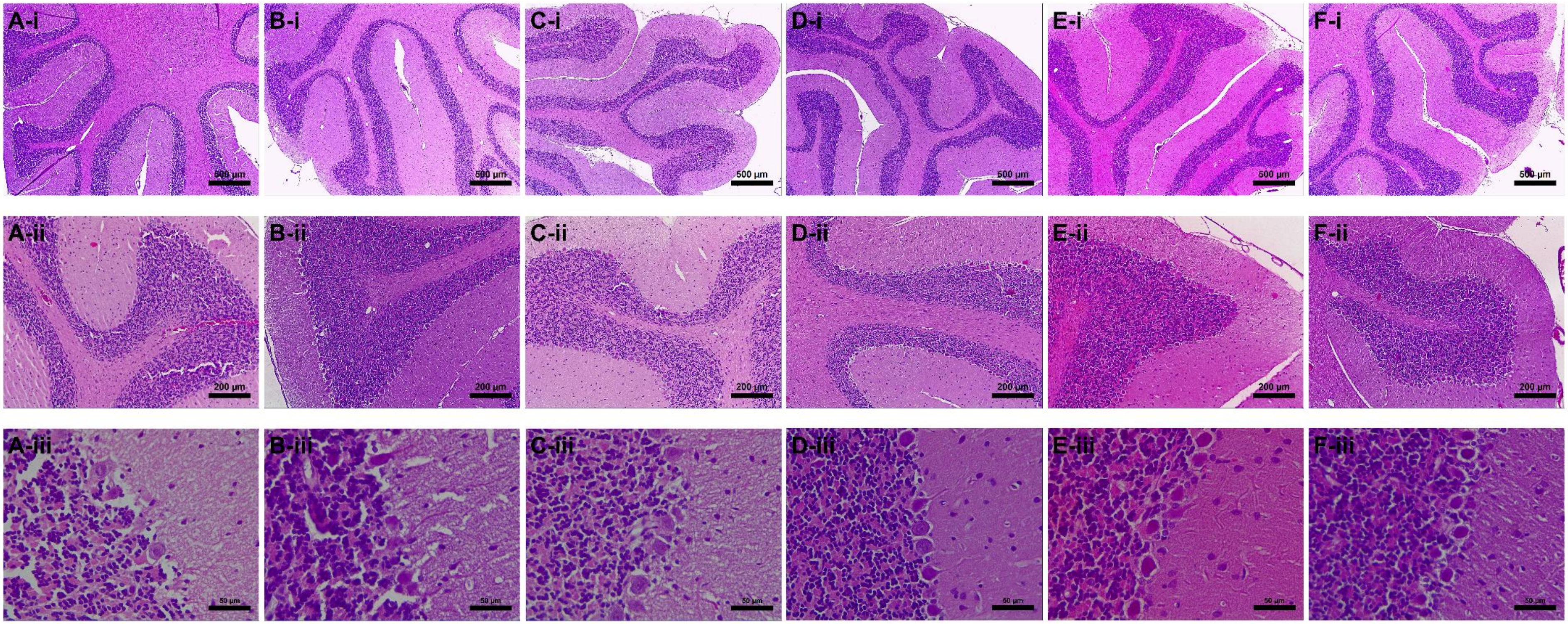

Figure S8 – Representative optical images of the histological sections of the cerebellum, at 40x (**i**), 100x (**ii**), and 400x magnification (**iii**) stained with hematoxylin-eosin, of non- (Sham, **A**) and carrageenan-induced knee inflammation in rats treated with PBS (saline, **B**), DE-R (3.5 µg/mL of alkylamides 8/9, **C**), FLPs (0.4 mM of PC, **D**), FLPs + DE-R (3.5 µg/mL of alkylamides 8/9 and 0.4 mM of PC, **E**), and celecoxib (10 µM, **F**) at 10th day.

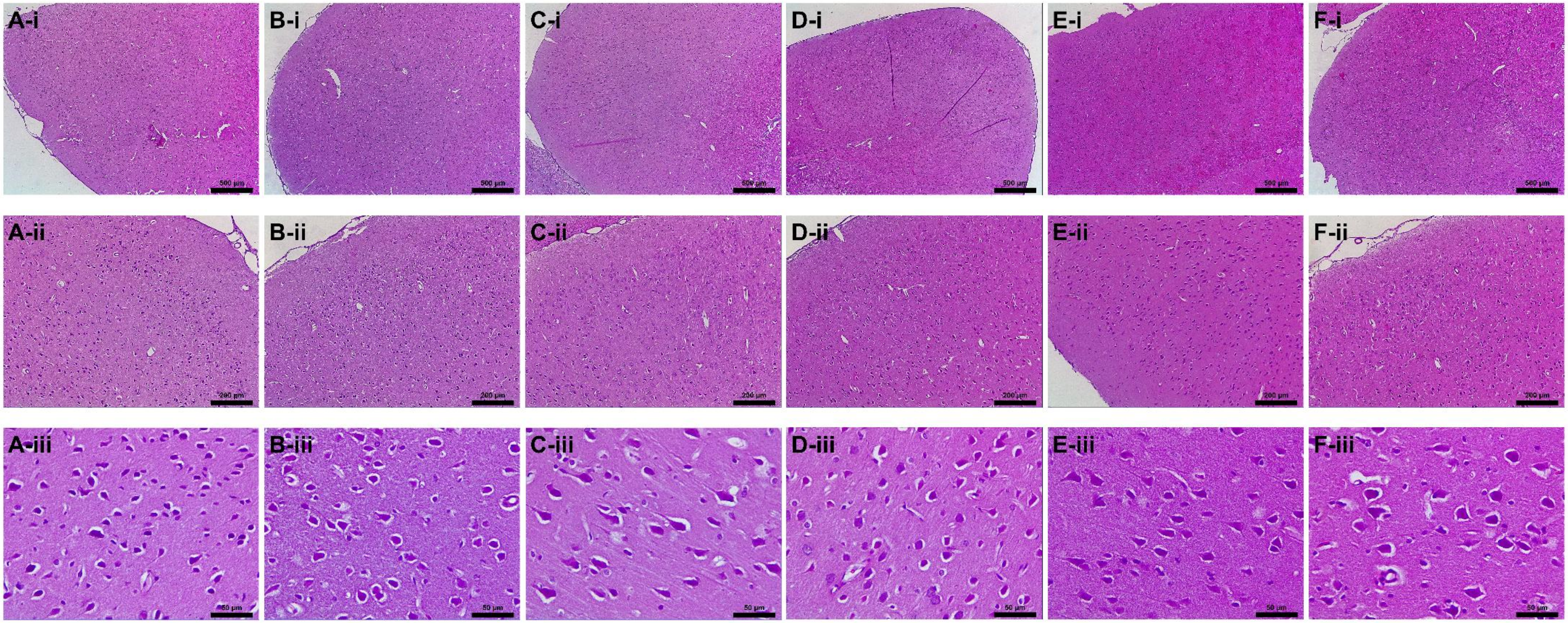

Figure S9 – Representative optical images of the histological sections of the cerebral cortex, at 40x (**i**), 100x (**ii**), and 400x magnification (**iii**) stained with hematoxylin-eosin, of non- (Sham, **A**) and carrageenan-induced knee inflammation in rats treated with PBS (saline, **B**), DE-R (3.5 µg/mL of alkylamides 8/9, **C**), FLPs (0.4 mM of PC, **D**), FLPs + DE-R (3.5 µg/mL of alkylamides 8/9 and 0.4 mM of PC, **E**), and celecoxib (10 µM, **F**) at 10th day.

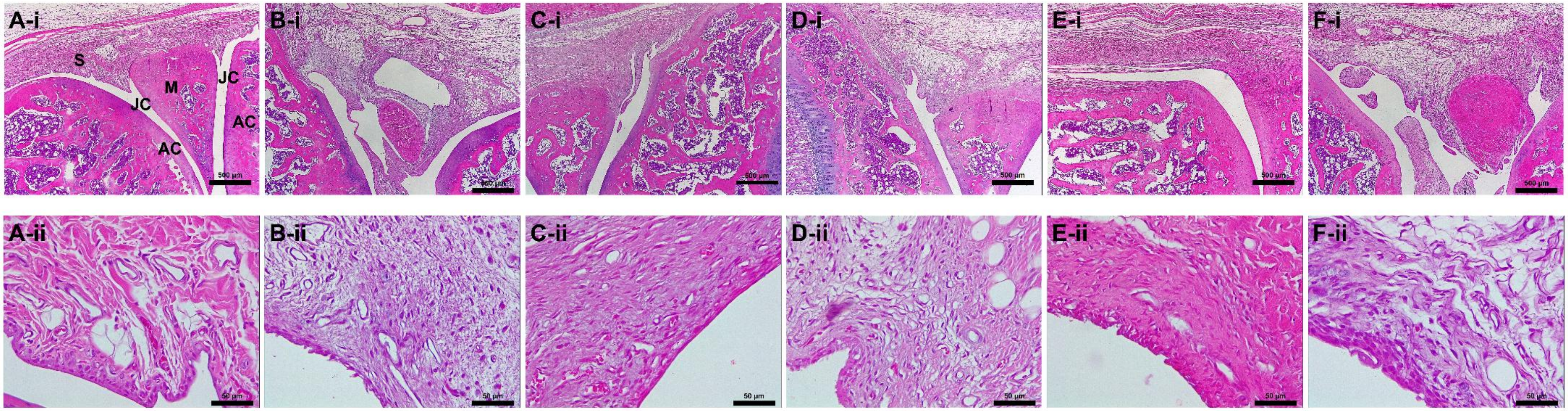

Figure S10 – Representative optical micrographs of the histological sections of the knee joint, at 40x (**i**) and 400x magnification (**ii**) stained with hematoxylin-eosin, of non- (Sham, **A**) and) and carrageenan-induced knee inflammation in rats treated with PBS (saline, **B**), DE-R (3.5 µg/mL of alkylamides 8/9, **C**), FLPs (0.4 mM of PC, **D**), FLPs + DE-R (3.5 µg/mL of alkylamides 8/9 and 0.4 mM of PC, **E**), and celecoxib (10 µM, **F**) at 10th day. S: synovial membrane; M: meniscus; JC: joint cavity; AC: articular cartilage.

References

[1] N.B. Cech, M.S. Eleazer, L.T. Shoffner, M.R. Crosswhite, A.C. Davis, A.M. Mortenson, High performance liquid chromatography/electrospray ionization mass spectrometry for simultaneous analysis of alkamides and caffeic acid derivatives from *Echinacea purpurea* extracts, J. Chromatogr. A. 1103 (2006) 219–228. doi:10.1016/j.chroma.2005.11.008.

[2] K. Spelman, M.H. Wetschler, N.B. Cech, Comparison of alkylamide yield in ethanolic extracts prepared from fresh versus dry *Echinacea purpurea* utilizing HPLC–ESI-MS, J. Pharm. Biomed. Anal. 49 (2009) 1141–1149. doi:10.1016/j.jpba.2009.02.011.

[3] F. Pellati, F. Epifano, N. Contaldo, G. Orlandini, L. Cavicchi, S. Genovese, D. Bertelli, S. Benvenuti, M. Curini, A. Bertaccini, M.G. Bellardi, Chromatographic methods for metabolite profiling of virus- and phytoplasma-infected plants of *Echinacea purpurea*, J. Agric. Food Chem. 59 (2011) 10425–10434. doi:10.1021/jf2025677.

[4] M.O. Thomsen, X.C. Fretté, K.B. Christensen, L.P. Christensen, K. Grevsen, Seasonal variations in the concentrations of lipophilic compounds and phenolic acids in the roots of *Echinacea purpurea* and *Echinacea pallida*, J. Agric. Food Chem. 60 (2012) 12131–12141. doi:10.1021/jf303292t.

[5] X. He, L. Lin, M.W. Bernart, L. Lian, Analysis of alkamides in roots and achenes of *Echinacea purpurea* by liquid chromatography-electrospray mass spectrometry, J. Chromatogr. A. 815 (1998) 205–211. doi:10.1016/S0021-9673(98)00447-6.

[6] E. Mudge, D. Lopes-Lutz, P. Brown, A. Schieber, Analysis of alkylamides in Echinacea plant materials and dietary supplements by ultrafast liquid chromatography with diode array and mass spectrometric detection, J. Agric. Food Chem. 59 (2011) 8086–8094. doi:10.1021/jf201158k.

[7] R. Bauer, P. Remiger, TLC and HPLC analysis of alkamides in Echinacea drugs, Planta Med. 55 (1989) 367–371. doi:10.1055/s-2006-962030.
